# RasGRP4 aggravates ischemia-reperfusion injury in diabetic kidneys by mediating communication between macrophages and T cells

**DOI:** 10.1172/jci.insight.187653

**Published:** 2024-12-10

**Authors:** Li Zhang, Zhanglong Wang, Yunqi Wu, Binshan Zhang, Zhongli Wang, Sisi Chen, Xuying Meng, Pei Yu, Saijun Zhou

**Affiliations:** 1 NHC Key Lab of Hormones and Development and Tianjin Key Lab of Metabolic Diseases,Tianjin Medical University Chu Hsien-I Memorial Hospital & Institute of Endocrinology, Tianjin, China.

**Keywords:** Endocrinology, Nephrology, Diabetes, Macrophages, T cells

## Abstract

Diabetes mellitus (DM) is acknowledged as an independent risk factor for acute kidney injury. Ras guanine nucleotide-releasing protein-4 (*RasGRP4*) exerts a notable role in modulating immune-inflammatory responses and kidney disease progression in diabetes. Herein, we delved into the specific role and mechanism of *RasGRP4* in diabetic renal ischemia-reperfusion injury. Diabetes was induced by a high-fat diet and streptozocin (STZ) injections, followed by creating an ischemia-reperfusion kidney injury via renal pedicle clamping and reperfusion. In vitro, a high glucose and hypoxia-reoxygenation modeled cellular inflammatory injury. We found *RasGRP4*-KO mice, compared with C57BL/6J (WT) mice, showed markedly less renal dysfunction and fibrosis in diabetic ischemia-reperfusion injury. There was a significant decrease in the renal infiltration of M1 macrophages and Th17 cells, along with downregulated IL-17 pathway proteins and effectors. In vitro, *RasGRP4* deletion restrained M1 macrophage polarization and Th17 cell differentiation, inhibiting the IL-17 signaling pathway in HK-2 cells. Hyperglycemia intensified renal inflammation state. Together, *RasGRP4*, through the regulation of interactions among M1 macrophages, CD4^+^ T cells, and HK-2 cells, formed a cascade that intensified the inflammatory storm activity, ultimately exacerbating the inflammatory injury of diabetic ischemia-reperfusion kidneys. DM intensified this inflammatory injury mechanism, worsening the injury from renal ischemia-reperfusion.

## Abstract 2

<strong>A cascade of exacerbated inflammation, centered around M1 macrophages, Th17 cells, and HK-2 cells, which was mediated by RasGRP4, aggravating diabetic ischemia-reperfusion renal injury</strong><strong> progression</strong>

## Graphical abstract

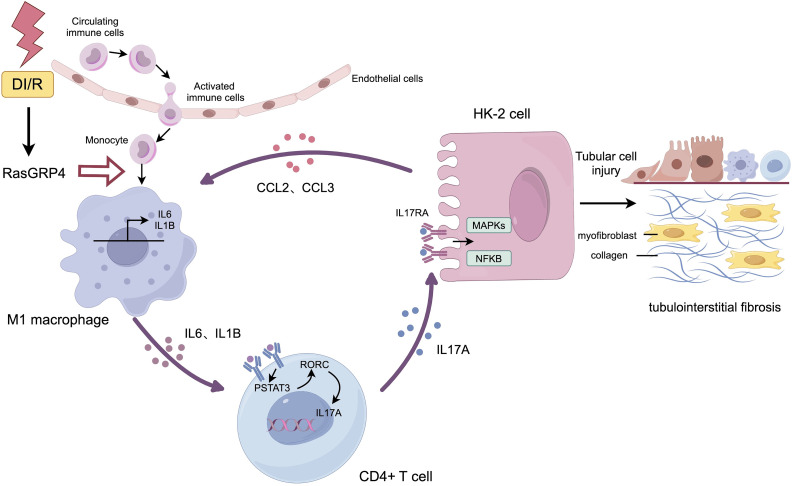

## Introduction

Acute kidney injury (AKI) and diabetes mellitus (DM) have emerged as substantial public health challenges, exerting a socioeconomic burden globally. Recent shifts in the etiology of AKI highlight the growing body of evidence implicating DM as an autonomous risk factor in the onset of AKI (1, 2). Concurrently, an alternative view posits that AKI may represent a genuine complication arising from DM (3). A pivotal study by Hapca et al. revealed that patients with diabetes, even without chronic kidney disease, exhibited an incidence of AKI that was 4.7-fold higher than that observed in their nondiabetic peers (4). Moreover, individuals with type 2 DM (T2DM) exhibit a more precipitous decline in eGFR prior to the onset of AKI compared with their nondiabetic counterparts (4).

In a chronic hyperglycemic state, inadequate metabolic regulation can permanently affect the progression of renal disease. Diabetic kidneys typically present with decreased oxygen supply, vascular injury, and intensified inflammatory reactions, thereby augmenting the kidneys’ vulnerability to hypoxic conditions (1). Inflammation represents a key factor in the pathogenesis of AKI. After AKI, both resident and infiltrating immune cells orchestrate the renal inflammatory milieu (5, 6). When concurrent with diabetes, hyperglycemia can serve as an ideal substrate that fosters a proinflammatory environment, exacerbating the inflammatory state of AKI. Despite the highly complex pathophysiological mechanisms linking DM and AKI, few studies have specifically discussed the role of diabetes in AKI, and the processes by which diabetes aggravates AKI, thus, remain to be further explored.

As a leading driver of AKI in clinical practice, ischemia-reperfusion injury (IRI) is principally characterized by tubular epithelial cell injury and demise (7). As the main participants in the IRI inflammatory response, macrophages will significantly increase within 24 hours after reperfusion (8). The activation and functionality of macrophages are contingent upon the phase of tissue damage and subsequent repair processes. Signals from the damaged renal microenvironment are instrumental in modulating the activation state of monocytes and macrophages. Following ischemia, the initial phase of reperfusion is characterized by the emission of damage-associated molecular patterns from necrotic or compromised tubular cells, reactive oxygen species generation, and neutrophils infiltration. These elements collectively contribute to the priming of resident mononuclear phagocytes and the polarization of infiltrating monocytes into proinflammatory macrophages (7). T cells, integral to the adaptive immune system, serve as a link between innate and adaptive immunity (9), which are also important participants in renal IRI injury. The products of T cells are essential for the induction of the expression of endothelial cell adhesion molecule, facilitating the recruitment and activation of macrophages (10). These inflammatory cascade reactions can aggravate renal injury and the sharp deterioration of renal function.

The IL-17–driven metabolic pathway conveys essential signals for tissue homeostasis (11). IL-17A is a pleiotropic cytokine predominantly released by Th17 (12). Extensive research has implicated Th17 cells and IL-17A in the pathogenesis of a spectrum of renal disorders (13–15).

The guanine nucleotide exchange factor family encompasses 4 members including Ras guanine nucleotide-releasing protein 1–4 (RasGRP1–4), all of which are regulated by Ca^2+^ and diacylglycerol (16). Among them, *RasGRP4* serves as a specific activator of Ras proteins, predominantly expressed in immune cells where it contributes to inflammatory responses by regulating the proliferation and migration of these cells (17). We previously found that *RasGRP4* can promote the secretion of the inflammatory factor IFN-γ by mediating cross-communication between CD117^+^ NK cells and DCs (18). Furthermore, the KO of *RasGRP4* can reduce macrophages and CD3^+^ T cell infiltration and alleviate inflammatory injury in diabetic kidney disease (DKD) model mice (19).

In our current investigation, a notable upregulation of renal *RasGRP4* expression was observed in diabetic mice following IRI, which was confirmed in a model system in which macrophages were treated with high glucose (HG) combined with hypoxia/reoxygenation (H/R). This observation has led us to hypothesize a potentially vital role for *RasGRP4* in the context of diabetic renal IRI. To explore this hypothesis, we utilized *RasGRP4*-KO mice to assess the effect of *RasGRP4* on diabetic ischemia-reperfusion renal injury (DI/R). In addition, the effect of *RasGRP4* on the interaction among macrophages, CD4^+^ T cells, and tubular epithelial cells and the underlying mechanisms were further explored in cell-based experiments.

## Results

### RasGRP4 was upregulated in DI/R mice renal and affected metabolic and biochemical indices in DI/R and nondiabetic ischemia-reperfusion (NI/R) mice.

**Figure 1:**
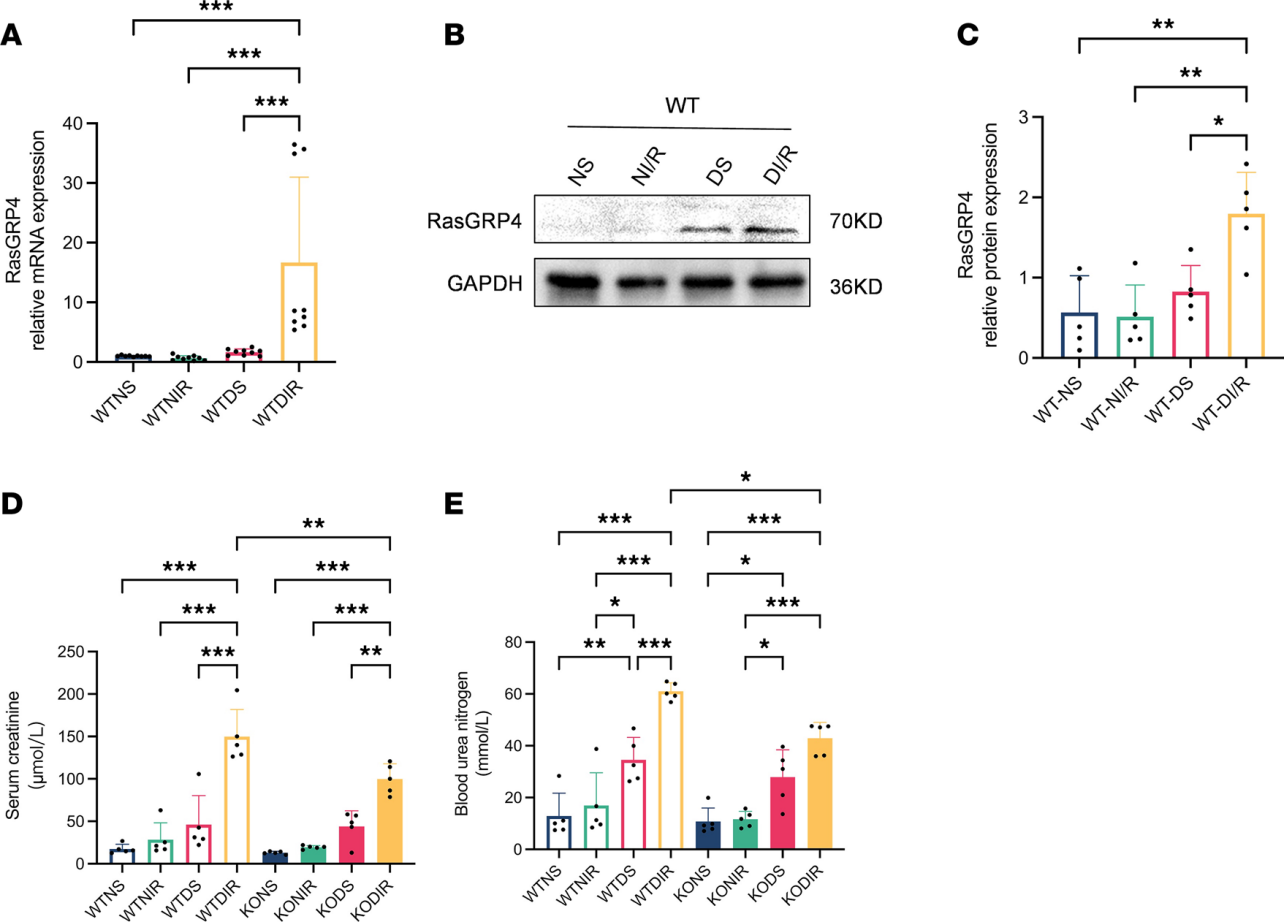
*RasGRP4* was upregulated in DI/R group and exacerbated the renal function impairment. (**A**) qPCR to detect the mRNA expression level of *RasGRP4* in the kidneys (*n* = 3/group). (**B** and **C**) Western blot to detect the protein expression level of *RasGRP4* in renal tissues and quantitative analysis (*n* = 5/group). (**D**) Serum creatinine levels (*n* = 5/group). (**E**) Urea nitrogen levels (*n* = 5/group). *P < 0.05, **P < 0.01, ***P < 0.001. *P* values were calculated using 1-way ANOVA test.

Given our previous findings that knocking out *RasGRP4* in DKD mice reduces macrophage and CD3^+^ T cell infiltration, thereby alleviating renal inflammatory damage (19), we sought to explore whether *RasGRP4* is also a harmful factor in other diabetic-related diseases and to clarify the underlying regulatory mechanisms. We established a T2DM model utilizing a high-fat diet in conjunction with i.p. streptozocin (STZ) injection, and an IRI kidney injury model was induced through the clamping of renal pedicles followed by reperfusion. Our results demonstrate a notable increase in the expression of *RasGRP4* at both the transcriptional and translational levels in DI/R mice (Figure 1, A–C). Further modeling in WT and KO mice was thus conducted to investigate the functional role of *RasGRP4* in DI/R condition.

In contrast to the nondiabetic group mice, diabetic mice exhibited a significant elevation in weight, blood glucose, and total cholesterol. Nevertheless, there were no substantial differences detected between WT diabetic mice and KO diabetic mice (Table 1 and Supplemental Figure 1, A and B). Serum biochemical tests showed that the alanine aminotransferase and aspartate aminotransferase levels in DI/R mice were significantly higher than those in nondiabetic sham-operated (NS) mice, diabetic sham-operated (DS) mice, and NI/R mice. Compared with the WTDI/R (WT diabetic ischemia-reperfusion renal injury) group, the KODI/R (*RasGRP4*-KO diabetic ischemia-reperfusion renal injury) group exhibited a smarkedly reduced level of serum creatinine (Scr) (*P* = 0.01) and blood urea nitrogen (BUN) levels (*P* = 0.02). Furthermore, renal function was considerably compromised in the DI/R group when compared with the NI/R group (Figure 1, D and E). These outcomes suggest that the KO of the *RasGRP4* was sufficient to significantly alleviate renal functional damage in DI/R mice, and hyperglycemia significantly aggravated the liver and kidney function damage in IRI mice.

### Effect of RasGRP4 on renal tubule injury and renal fibrosis in DI/R mice.

**Figure 2:**
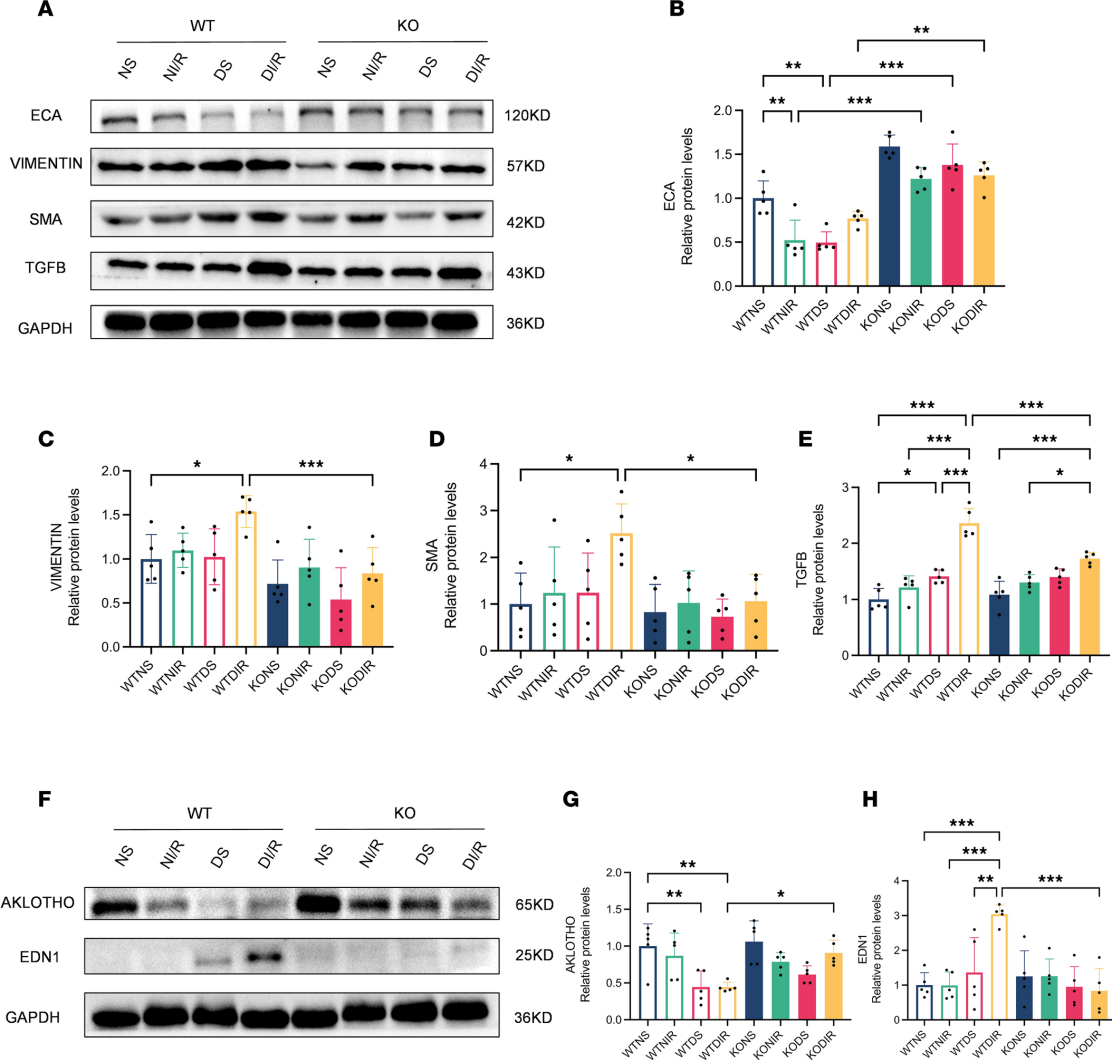
*RasGRP4* exacerbated renal tubular injury and renal fibrosis in diabetic ischemia-reperfusion injury kidneys. (**A**–**E**) Western blot to detect the expression levels and quantitative analysis of ECA, VIMENTIN, SMA, and TGF-β proteins in renal tissue (*n* = 5/group). (**F**–**H**) Western blot to detect the expression levels of AKLOTHO and EDN1 proteins in renal tissues (*n* = 5/group). *P < 0.05, **P < 0.01, ***P < 0.001. *P* values were calculated using 1-way ANOVA test.

Renal fibrosis, as the pathological basis and common endpoint in the progression of chronic kidney disease toward end-stage renal disease, is a critical determinant in the deterioration of renal function. Tubular injury serves as the central link in IRI. To observe the effect of *RasGRP4* on renal tissues, we performed pathological staining. Through this approach, we discovered that the WTDI/R group exhibited more severe renal tubular lumen dilation compared with the KODI/R group, with shedding of tubular epithelial cells, the formation of a bare tubule basement membranes, and the appearance of apoptotic tubular cells compressed into triangular shapes, along with abundant glycogen deposition and collagen fiber proliferation (Supplemental Figure 1C). By detecting the levels of the fibrosis-related proteins SMA, ECA, TGF-β, and VIMENTIN, as well as the renal tubular injury–related factors AKLOTHO and EDN1, our results reveal that the extent of renal fibrosis and tubular injury in the WTDI/R mice was more severe than in the KODI/R mice (Figure 2). Concurrently, DM was able to partially exacerbate the occurrence of IRI-induced tubular injury and renal fibrosis. These results indicate that DM can aggravate pathological renal damage in IRI mice, while knocking out *RasGRP4* can alleviate renal tubular injury and fibrosis in DI/R mice.

### Effect of RasGRP4 on the Th17 immune response in DI/R mice.

**Figure 3:**
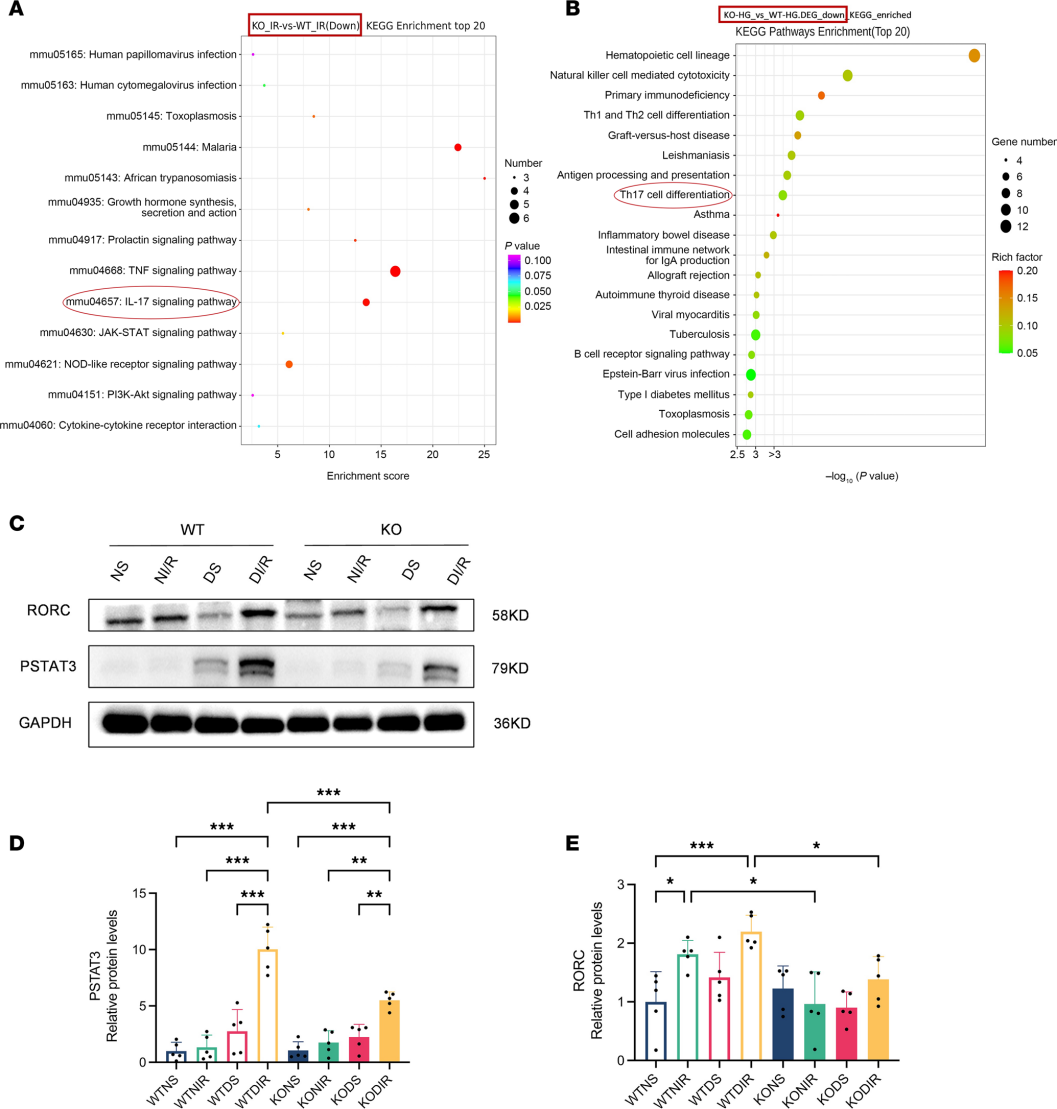
*RasGRP4* promoted the differentiation of CD4^+^ T cells to Th17 cells in the kidneys of diabetic ischemia-reperfusion injury. (**A**) The top 20 bubble chart of KEGG enrichment of differentially expressed genes in the ischemia-reperfusion kidney injury. (**B**) The top 20 bubble chart of KEGG enrichment of differentially expressed genes in the inflammatory injury model of peripheral blood mononuclear cells cultured in high glucose medium. (**C**–**E**) Western blot to detect the expression levels and quantitative analysis of PSTAT3 and RORC proteins in renal (*n* = 5/group). *P < 0.05, **P < 0.01, ***P < 0.001. *P* values were calculated using 1-way ANOVA test.

**Figure 4:**
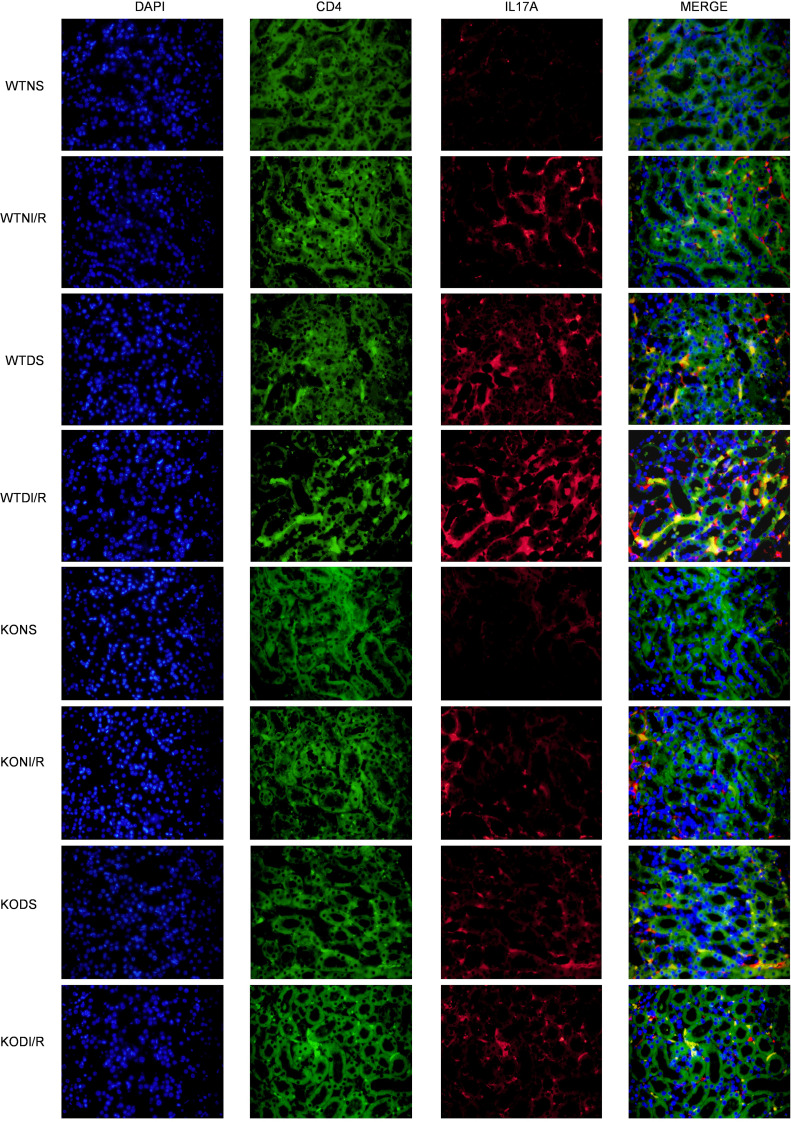
*RasGRP4* promoted infiltration of Th17 cells in the kidneys of diabetic ischemia-reperfusion injury. Immunofluorescence staining of CD4 and IL-17 in renal tissues. Total original magnification x400.

Previously, we established that *RasGRP4* was significantly involved in the differentiation of Th17 cells (*P* = 0.0002) and the IL-17 signaling pathway (*P* = 0.0017) through a transcriptomic approach (Figure 3, A and B). To delve deeper into the precise mechanisms whereby *RasGRP4* modulates DI/R injury, we performed IHC and multiplex immunofluorescence staining for the Th17 cell–specific markers IL-17A and CD4. We observed a higher infiltration of Th17 cells in the kidneys of WTDI/R mice as compared with KODI/R mice (Figure 4 and Supplemental Figure 2A). Simultaneously, the renal protein expression levels of the key Th17-associated transcription factors PSTAT3 and RORC in the WTDI/R mice were markedly elevated relative to those in the KODI/R mice (Figure 3, C–E). In comparison with the nondiabetic groups, the infiltration and expression of these Th17 transcription factors also tended to increase after IRI in DM. These results indicate that hyperglycemia can lead to aggravated Th17 cell infiltration in IRI mice, while knocking out *RasGRP4* could significantly suppressed the infiltration and activation of Th17 cells.

### Effect of RasGRP4 on macrophage polarization.

**Figure 5:**
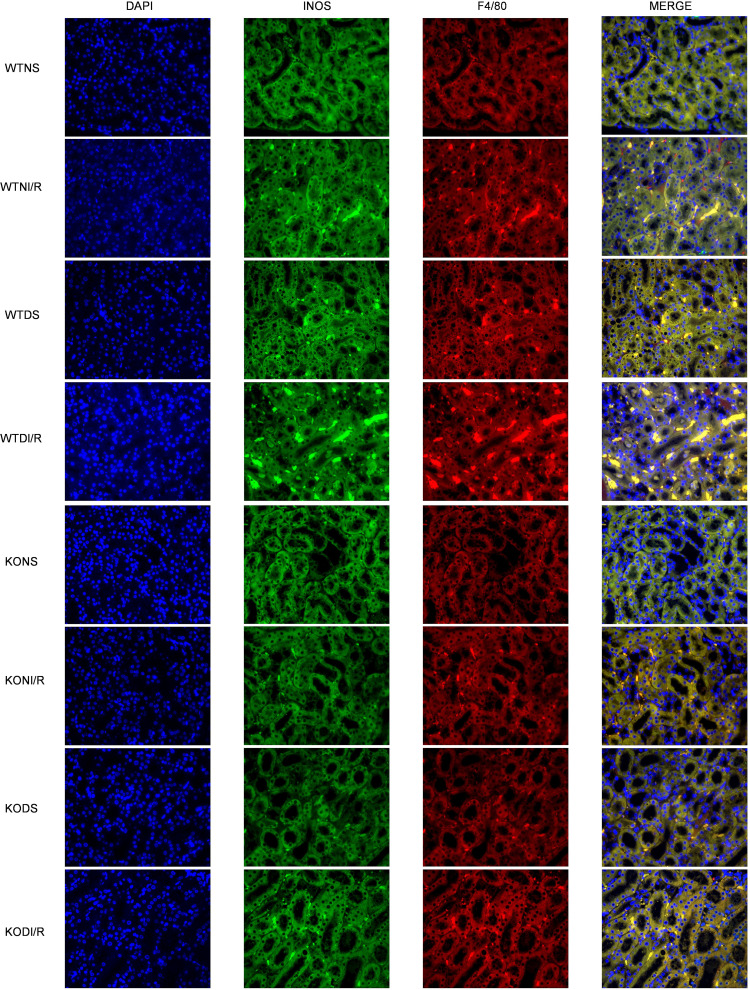
*RasGRP4* promoted infiltration of M1 macrophages in diabetic ischemia-reperfusion injury kidneys. Immunofluorescence staining of F4/80 and INOS in kidneys. Total original magnification x400/

**Figure 6:**
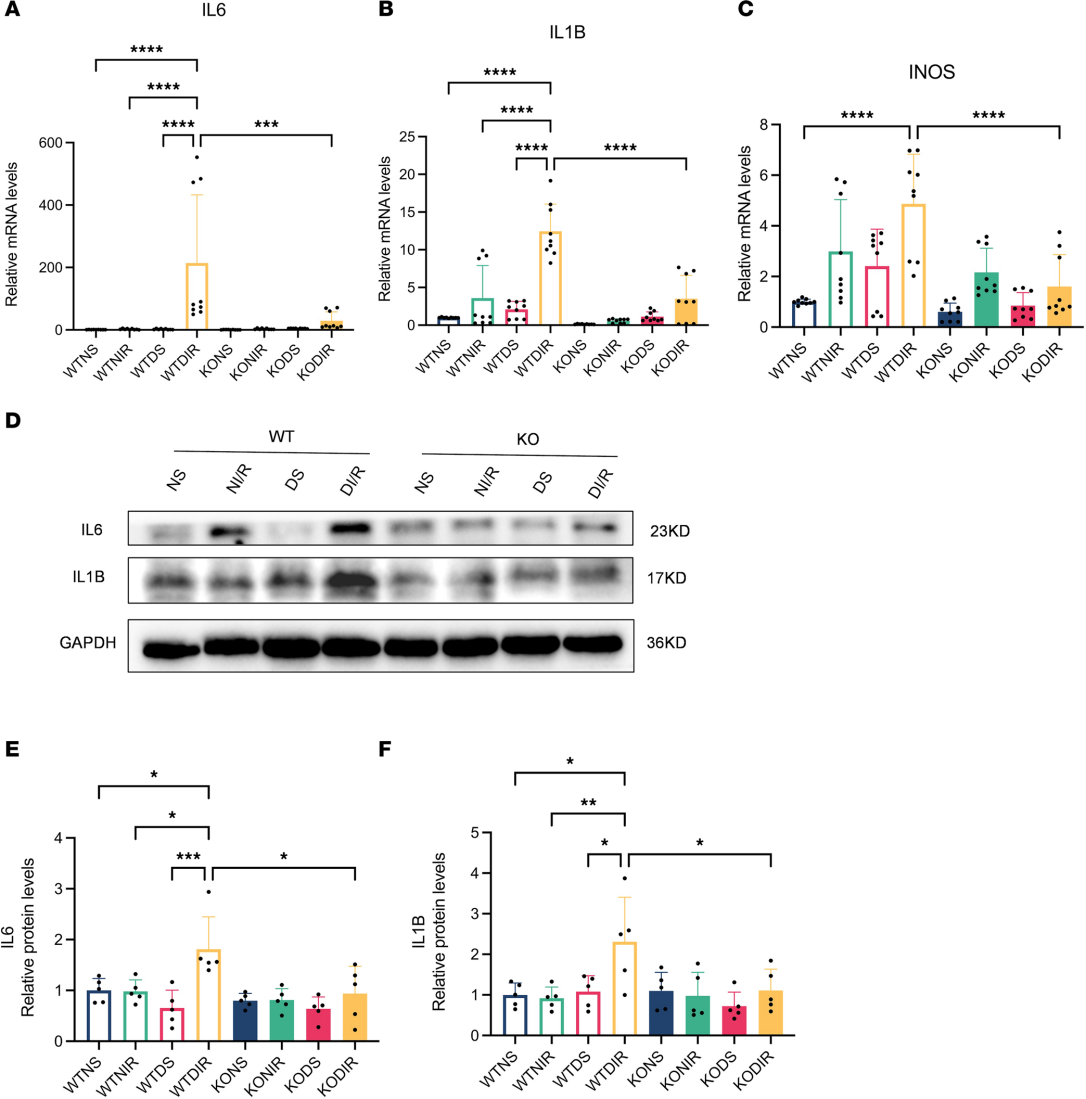
*RasGRP4* facilitated the polarization of macrophages toward M1 macrophages in diabetic ischemia-reperfusion injury kidneys. (**A**–**C**) qPCR for detecting the mRNA expression levels of *IL6*, *IL1B*, and *INOS* in kidneys (*n* = 3/group). (**D**–**F**) Western blot for detecting the protein expression levels of IL-6 and IL-1B in renal tissues and quantitative analysis (*n* = 5/group). *P < 0.05, **P < 0.01, ***P < 0.001, ****P < 0.0001. *P* values were calculated using 1-way ANOVA test.

The above results indicated that *RasGRP4* plays a pivotal regulatory role in the Th17 immune response. During T cell differentiation, M1 macrophages are known for their importance with respect to antigen presentation and activation. In addition, M1 macrophages, as primary mediators of the inflammatory process, have been found to be recruited in the early stages of kidney damage during AKI episodes (1). In this study, we found that the M1 macrophages infiltration (Figure 5 and Supplemental Figure 2B) and the pertinent inflammatory factor secretion (Figure 6) in the WTDI/R mice were notably enhanced compared with the KODI/R mice. Diabetes was thereby affiliated with the M1 macrophages recruitment and the expression of IL-6 and IL-1B during the IRI procedure. These outcomes indicated that the ablation of *RasGRP4* could restrain the infiltration and activation of M1 macrophages in the kidneys in settings of diabetic IRI, and hyperglycemia can readily engender a proinflammatory milieu.

### Effect of RasGRP4 on the IL-17 signaling pathway activation in the kidney of ischemia-reperfusion model mice.

**Figure 7:**
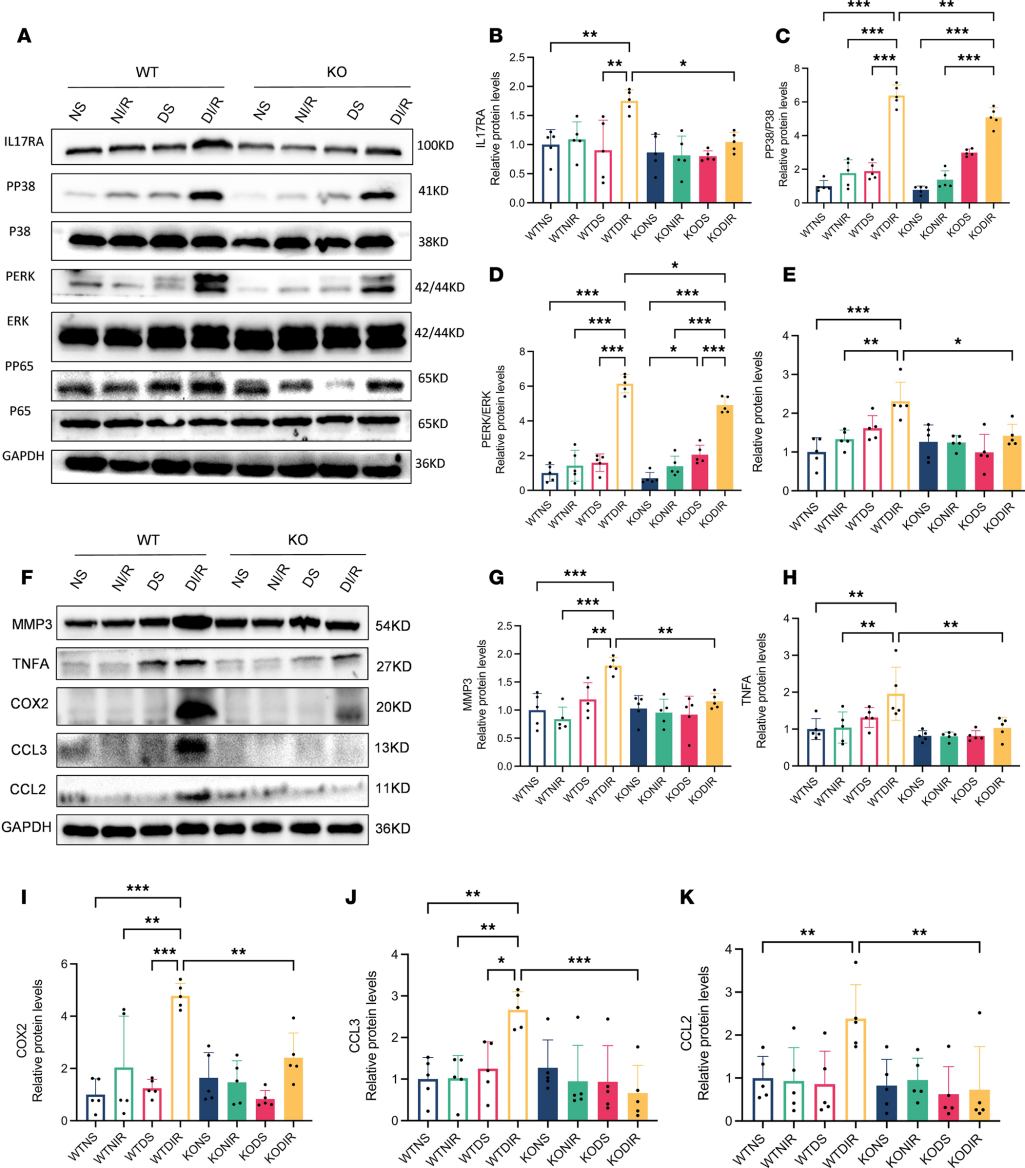
*RasGRP4* promoted the activation of the IL-17 signaling pathway in the kidneys with diabetic ischemia-reperfusion injury. (**A**–**E**) The protein expression levels and quantitative analyses of IL-17RA, PP38/P38, PERK/ERK, and PP65/P65 in renal tissue (*n* = 5/group). (**F**–**K**) The protein expression levels and quantitative analyses of MMP3, TNFA, COX2, CCL3, and CCL2 in renal tissue (*n* = 5/group). *P < 0.05, **P < 0.01, ***P < 0.001. *P* values were calculated using 1-way ANOVA test.

We have verified the fact that *RasGRP4* can prominently facilitate both M1 macrophages and Th17 cells infiltration in the context of diabetic IRI kidney. The interplay between immune cells and renal intrinsic cells, along with the activation of inflammatory signaling pathways, constitutes a critical pathological mechanism implicated in the advancement of diabetic-related renal disorders. To further observe the effect triggered by the Th17 immune response, we examined the protein expression levels of pivotal molecules within the IL-17 signaling pathway, including IL-17RA, PP65/P65, PERK/ERK, and PP38/P38, as well the effector cytokines TNFA and COX2, the chemokines CCL2 and CCL3, and the tissue remodeling factor MMP3 (Figure 7). We discovered that these factors were markedly upregulated in WTDI/R mice relative to KODI/R mice and moderately upregulated relative to WTNI/R mice. These findings indicate that the KO of *RasGRP4* notably inhibited the IL-17 signaling pathway activation in the DI/R kidneys, thereby suppressing the expression of cytokines and chemokines and delaying the process of renal tissue remodeling, with diabetes serving as a risk factor for the aggravation of inflammatory damage.

### RasGRP4 was upregulated in macrophages cultured under high-glucose and H/R conditions and affected the differentiation of Th17 cells mediated by M1 macrophages.

**Figure 8:**
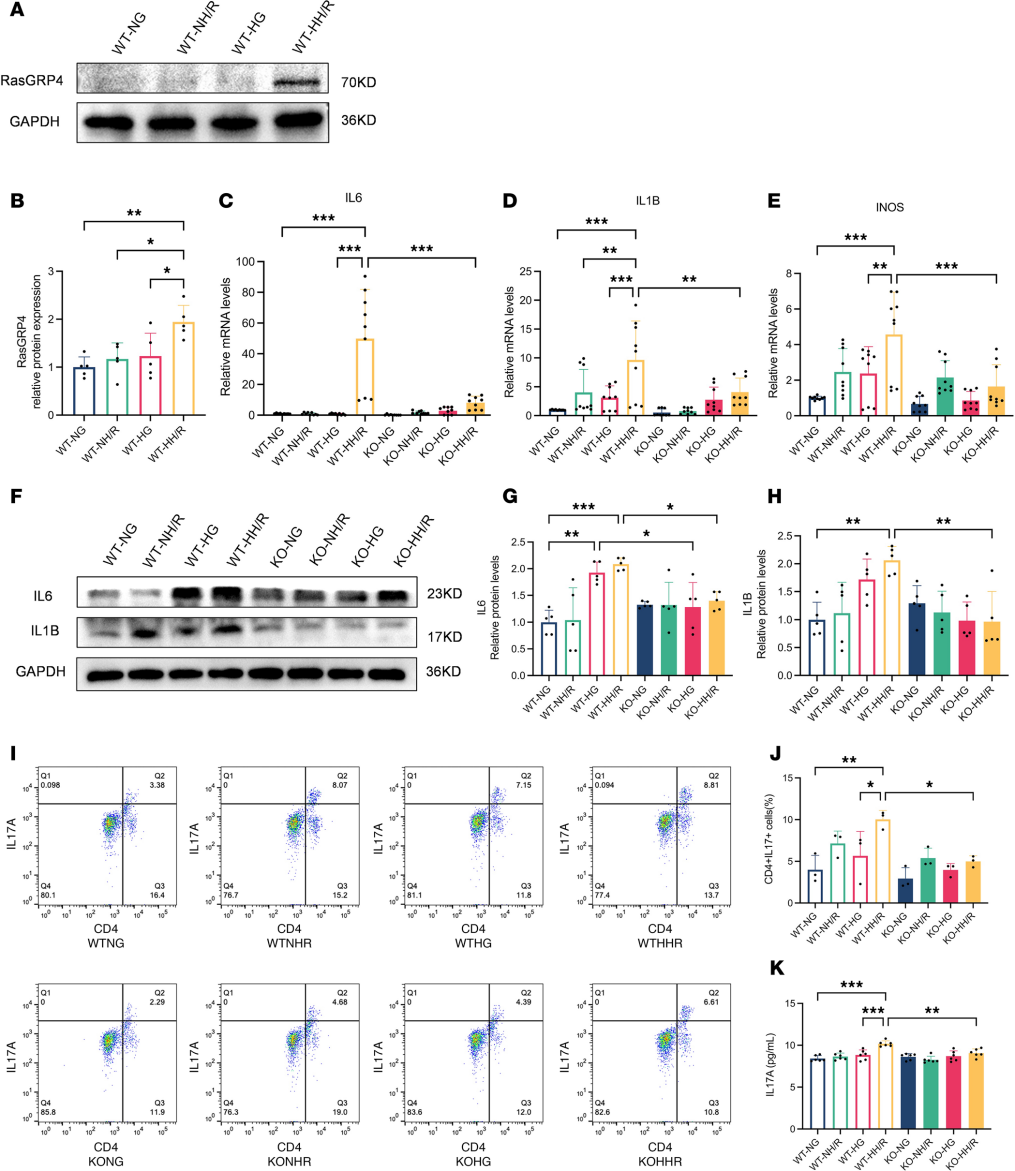
*RasGRP4* was upregulated in high glucose–cultured macrophages treated with H/R and affected the differentiation of Th17 cells mediated by M1 macrophages. (**A** and **B**) Western blot to detect the protein expression level of *RasGRP4* in peritoneal macrophages (*n* = 5/group). (**C**–**E**) qPCR to detect the mRNA expression levels of *IL6*, *IL1B*, and *INOS* in peritoneal macrophages (*n* = 3/group). (**F**–**H**) Western blot to detect the protein expression levels of IL-6 and IL-1B in peritoneal macrophages (*n* = 5/group). (**I** and **J**) Flow cytometric analysis of the influence on the differentiation ratio of CD4^+^IL17^+^(Th17) cells by peritoneal macrophages (*n* = 3/group). (**K**) ELISA to detect the content of IL-17A in the supernatant of the coculture system of macrophages and CD4^+^ T cells (*n* = 3/group). *P < 0.05, **P < 0.01, ***P < 0.001. *P* values were calculated using 1-way ANOVA test

Upon undergoing high-glucose culturing for 48 hours, succeeded by 4 hours of hypoxia and 2 hours of reoxygenation to establish a cellular model of inflammatory impairment, we ascertained that the *RasGRP4* protein expression in peritoneal-derived macrophages markedly augmented (Figure 8, A and B). The expressions of the M1 macrophage surface marker INOS and related proinflammatory factors IL-6 and IL-1B were conspicuously augmented (Figure 8, C–H), whereas this upregulation was markedly inhibited by the KO of *RasGRP4*. To further clarify whether *RasGRP4* adversely affected the interplay between macrophages and CD4^+^ T cells, the CD4^+^ T cells segregated from mice spleens were cocultured with peritoneal macrophages from the same species. By ascertaining the differentiation ratio of Th17 cells via flow cytometry and IL-17A content in the supernatants from the coculture system using ELISA kits, we found thatn, under conditions of high-glucose treatment combined with hypoxia and reoxygenation (HH/R), macrophages deriving from WT mice could foster the differentiation of CD4^+^ T cells into Th17 cells (Figure 8, I and J) and the secretion of IL-17A (Figure 8K), while the deletion of *RasGRP4* could significantly attenuate these effects.

**Figure 9:**
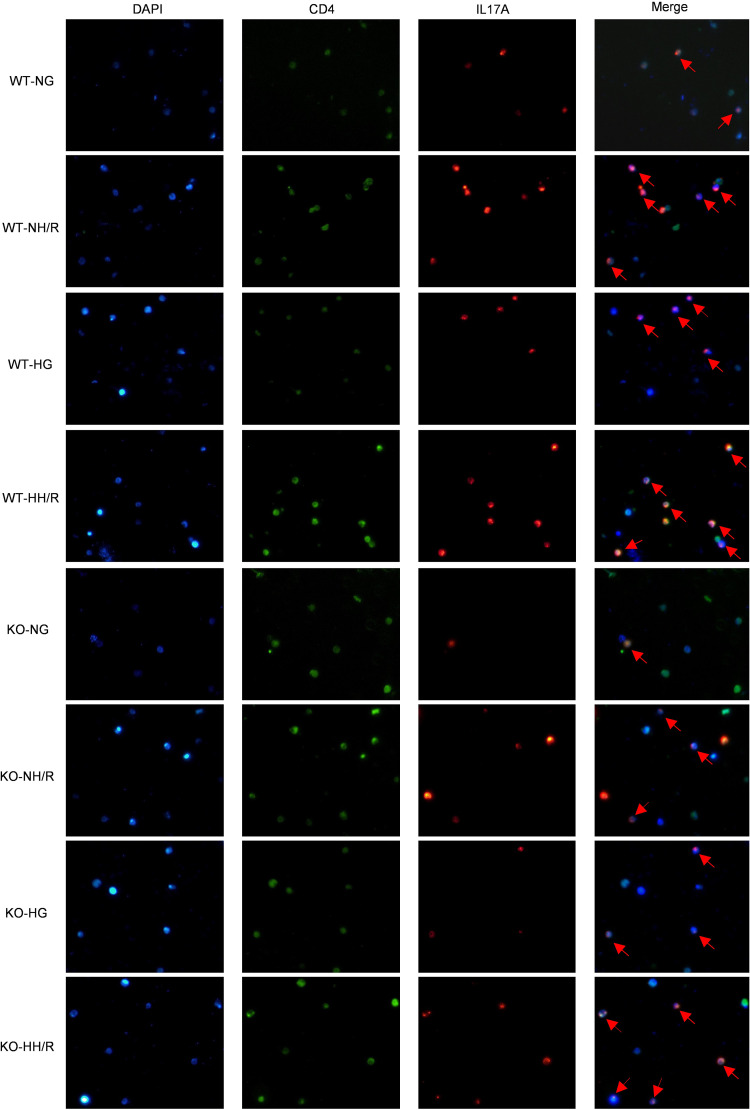
RasGRP4 promoted the differentiation of Th17 cells in peripheral blood mononuclear cells. Multiple immunofluorescence staining of CD4 and IL-17A. Total original magnification x400. Arrows indicate CD4^+^IL17^+^ (Th17) cells.

To further explore the interplay between immune cells and intrinsic renal cells as well as activation status of inflammatory signaling pathways, we employed murine PBMCs to simulate the colculture of macrophages and CD4^+^ T cells. PBMC is a comprehensive denomination for cells with a solitary nucleus existing in the peripheral blood, encompassing lymphocytes and monocytes. By performing multiplex immunofluorescence staining of PBMCs, we found that the IL-17A and CD4 costained area in the WT-HH/R mice was larger than that in the KO-HH/R mice (Figure 9), aligning with our previous findings. These results reveal that *RasGRP4* can promote the differentiation of macrophages into M1 macrophages, which promoted the differentiation of CD4^+^ T cells into Th17 cells and IL-17A secretion during HH/R injury; furthermore, the results reveal that high-glucose culture, to some extent, exacerbated the Th17 immune response under conditions of H/R injury.

### Effect of RasGRP4 on the IL-17 signaling pathway activation and its side effects in HK-2 cells cocultured with PBMCs under conditions of HH/R injury.

**Figure 10:**
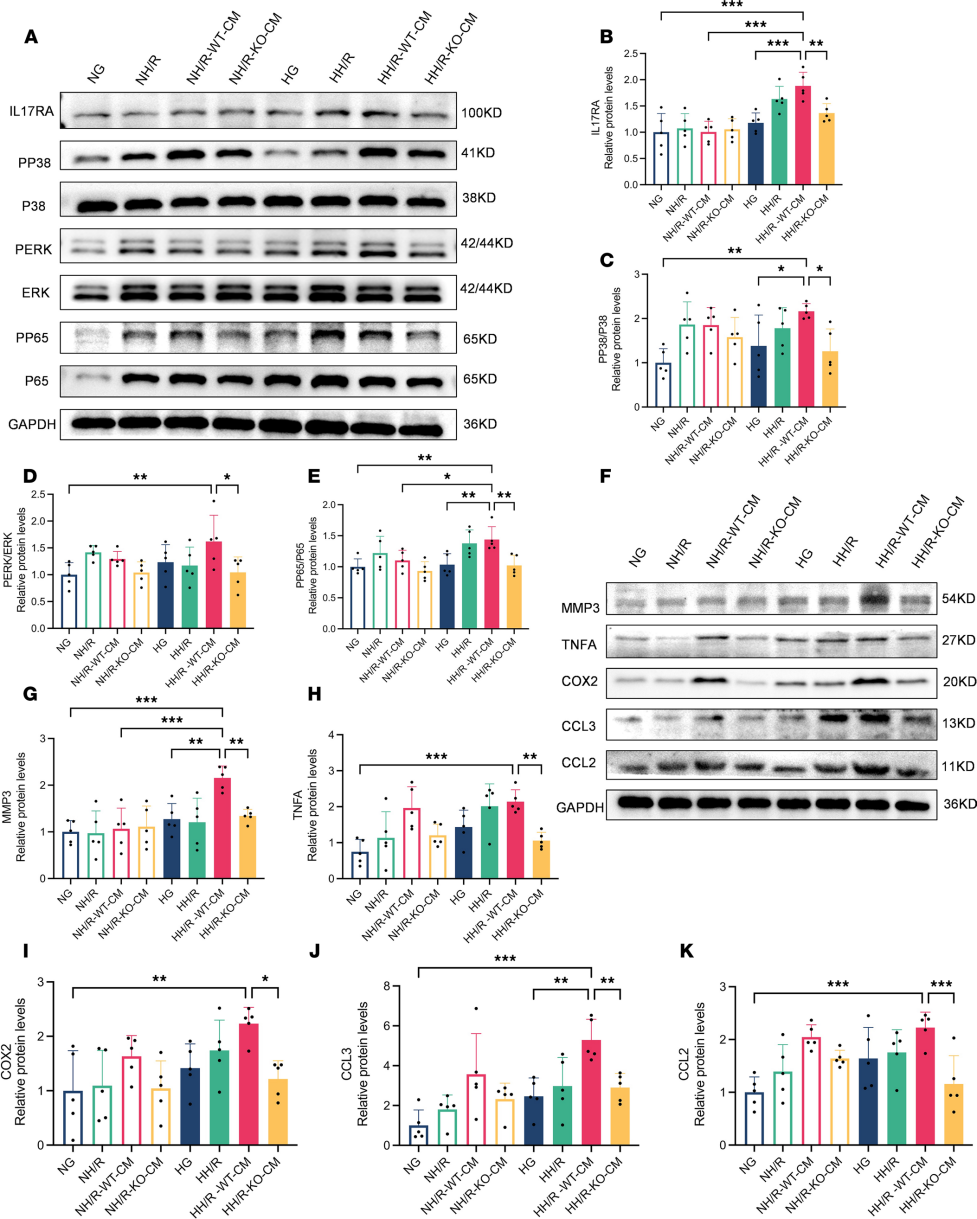
*RasGRP4* promoted the activation of the IL-17 signaling pathway in HK-2 cells cocultured with peripheral blood mononuclear cells under high glucose combined with hypoxia-reoxygenation injury. (**A**–**E**) Western blot to detect the protein expression levels and quantitative analyses of IL-17RA, PP38/P38, PERK/ERK, and PP65/P65 in HK-2 cells (*n* = 5/group). (**F**–**K**) Western blot to detect the protein expression levels and quantitative analysis of MMP3, TNFA, COX2, CCL3, and CCL2 in HK-2 cells (*n* = 5/group). *P < 0.05, **P < 0.01, ***P < 0.001, ****P < 0.0001. *P* values were calculated using 1-way ANOVA test.

Under HH/R conditions, the IL-17 signaling pathway activation and the effector factor expression in HK-2 cells cocultured with PBMCs from WT mice were significantly higher than those cocultured with PBMCs from KO mice (Figure 10).

**Figure 11:**
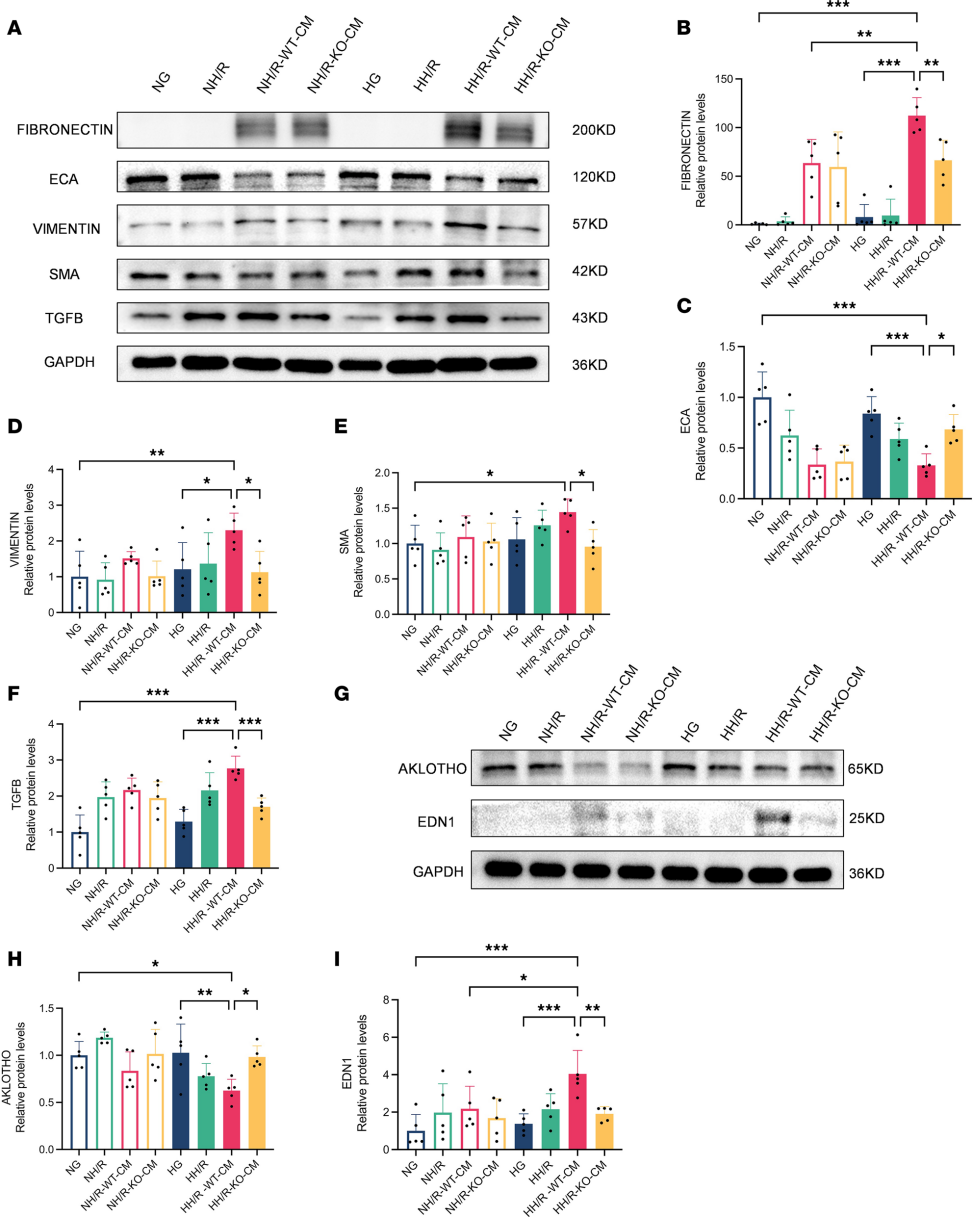
*RasGRP4* promoted the injury and fibrosis in HK-2 cells cocultured with peripheral blood mononuclear cells under high glucose combined with hypoxia-reoxygenation injury. (**A**–**F**) Western blot to detect the protein expression levels and quantitative analysis of FIBRONECTIN, ECA, VIMENTIN, SMA, and TGF-β in HK-2 cells (*n* = 5/group). (**G**–**I**) Western blot to detect the protein expression levels and quantitative analysis of AKLOTHO and EDN1 in HK-2 cells (*n* = 5/group). *P < 0.05, **P < 0.01, ***P < 0.001, ****P < 0.0001. *P* values were calculated using 1-way ANOVA test.

The maladaptive repair occurring due to injury is an important cause of cellular dysfunction. Hence, to explore the effect of the activation of immune cells and inflammation-related pathways on the extent of HK-2 cell injury and fibrosis under HH/R conditions, we discerned the expression of fibrosis-related proteins as well as tubular injury–related factors in HK-2 cells. The results indicate that, during HH/R injury, PBMCs from WT mice could promote tubular injury in HK-2 cells (Figure 11, G–I) and induce fibrosis (Figure 11, A–F), while the deletion of *RasGRP4* significantly alleviated these injurious effects, exerting a renoprotective role. These findings indicate that *RasGRP4* promoted IL-17 signaling pathway activation in HK-2 cells triggered by the interaction between M1 macrophages and CD4^+^ T cells during HH/R injury, accelerating the process of tubular injury and fibrosis, with hyperglycemia serving as an important factor that aggravated the immunoinflammatory injury of hypoxia-reoxygenated renal tubules.

### IL-17A inhibition partially attenuates the Th17 immune response and inflammatory kidney injury.

**Figure 12:**
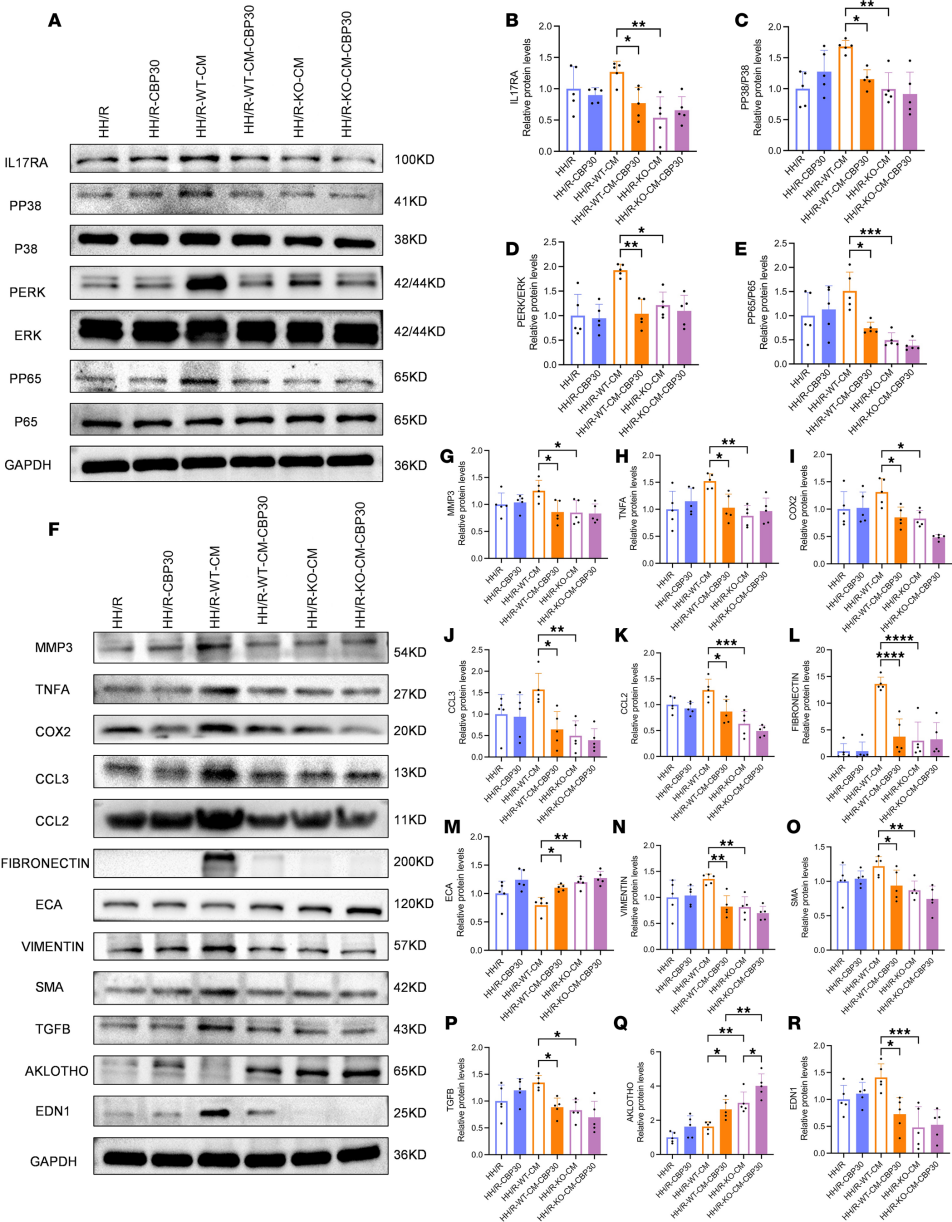
IL-17A inhibitor attenuated the activation of the IL-17 signaling pathway and inflammatory injury in HK-2 cells to a certain extent. (**A**–**E**) The protein expression levels and quantitative analyses of IL-17RA, PP38/P38, PERK/ERK, and PP65/P65 in HK-2 cells (*n* = 5/group). (**F**–**R**) The protein expression levels and quantitative analyses of MMP3, TNFA, COX2, CCL3, CCL2, ECA, VIMENTIN, SMA, TGF-β, AKLOTHO, and EDN1 in HK-2 cells (*n* = 5/group). *P < 0.05, **P < 0.01, ***P < 0.001, ****P < 0.0001. *P* values were calculated using 1-way ANOVA test.

To further verify the vital part played by the Th17 immune response and IL-17 pathway in DI/R, the IL-17A inhibitor SGC-CBP30 was incorporated into our experiments. The results show that there was a significant improvement in both Scr and BUN levels compared with the untreated group, indicating a partial restoration of renal function (Supplemental Figure 3). We found that SGC-CBP30 (also known as CBP30) suppressed the activation of the IL-17 pathway (Figure 12, A–E, and Supplemental Figure 4, C–F) and related inflammatory injury (Figure 12, F–R, and Supplemental Figure 4, G–H). In addition, the use of SGC-CBP30 also significantly inhibited the manifestation of the M1 macrophage–related inflammatory cytokines IL-6 and IL-1B, the key Th17 transcription factors PSTAT3 and RORC, and IL-17A in renal tissue (Supplemental Figure 4, A and B). This indicated that the Th17 immune response and IL-17 pathway play crucial roles in DI/R kidneys. In addition to having an effect on downstream HK-2 cells, IL-17A inhibition affects macrophages and Th17 cells. An inflammatory cascade reaction involving M1 macrophages, Th17 cells, and HK-2 cells may thus occur that aggravates DI/R injury–related disease processes.

## Discussion

*RasGRP4* serves as a central hub molecule in the proinflammatory pathway triggered by G-protein-coupled receptors (20). Our previous study delved into the intricate interactions between immune cells and renal intrinsic cells, implicating *RasGRP4* (18, 19). We discovered that under diabetic conditions, *RasGRP4* can — by influencing the infiltration of renal immune cells — regulate the interaction between mononuclear cells and glomerular vascular endothelial cells, thereby giving rise to the pathogenesis and advancement of DKD (19). Consequently, we sought to further investigate the potential involvement of *RasGRP4* in the regulatory mechanisms of diabetes-related kidney diseases and to elucidate its specific molecular mechanisms of activity. We observed significant upregulation of *RasGRP4* after renal IRI in diabetic mice, and this was further confirmed in the macrophage model under conditions of HH/R injury. The ablation of *RasGRP4* significantly mitigated the renal function decline, the tubular damage degree, and the renal fibrosis extent in DI/R mice. Thus, *RasGRP4* is posited as a critical player in diabetic renal IRI, underscoring the necessity for a deeper exploration of its mechanisms.

As one of the subsets of CD4^+^ Th lymphocytes, Th17 cells are involved in the pathogenesis of a multiplicity of autoimmune diseases and inflammatory states (21). The role of Th17 cells and their primary effector cytokine, IL-17A, in the genesis of diverse renal disorders has increasingly come under scrutiny (22). Our previous transcriptomic analyses have demonstrated a considerable regulatory influence of *RasGRP4* on Th17 cell differentiation and the activation of the IL-17 signaling cascade. To further confirm this result, in DI/R mice, through immunofluorescence and IHC, the genetic ablation of *RasGRP4* was observed to markedly curtail the infiltration of Th17 cells and significantly diminish the protein expression of the key Th17 transcription factors PSTAT3 and RORC. In the purview of T cell differentiation, M1 macrophages are instrumental mediators of antigen presentation and the subsequent activation of T cells. The antigens presented by M1 macrophages are identified by cognate T cell receptors. With adequate costimulation, this interaction triggers T cell activation and proliferation (23). A surge in macrophage numbers within the early phase following ischemia/reperfusion has been documented in prior studies (8). We further observed that KO of *RasGRP4* could significantly inhibit the M1 macrophage infiltration and the proinflammatory cytokines IL-6 and IL-1B expression in the DI/R group. To further explore the interplay between macrophages and Th17 cells, we primarily extracted macrophages from the abdominal cavity. Using a cellular inflammatory injury model established using the HH/R condition, the absence of *RasGRP4* was found to substantially restrain the activation of M1 macrophages. By coculturing peritoneal macrophages with CD4^+^ T cells sorted from the spleen, flow cytometry and ELISA approaches revealed that *RasGRP4* deletion could significantly reduce the Th17 differentiation ratio and inhibit IL-17A secretion by Th17 cells during HH/R injury.

The interplay between immune cells and inherent renal cells, as well as the activation of inflammatory signaling pathways, constitutes a important pathological mechanism implicated in the advancement of diabetic-related kidney diseases. IRI is traditionally defined as a tubular disease, with the impairment and demise of proximal tubular epithelial cells being the most prevalent pathological hallmarks, irrespective of the etiology (7). These injured tubular epithelial cells are instrumental in the propagation of inflammatory and fibrotic processes, secreting an array of chemokines that escalate the infiltration and aggregation of immune cells and amplify the inflammatory response within the kidney (24–26). Hence, investigating the mechanism of proximal tubular epithelial cell injury is of considerable importance for the prophylaxis and therapy of diabetic kidney IRI. In vivo, we discovered that knocking out *RasGRP4* could suppress the expression of the key molecules IL-17RA, PP38/P38, PERK/ERK, PP65/P65, TNFA, COX2, CCL2, CCL3, and MMP3 associated with the IL-17 signaling pathway. In vitro, considering the complexity and cost associated with the sorting of CD4^+^ T cells, and the low yield from this process, we employed PBMCs to simulate the coexistence system of macrophages and CD4^+^ T cells, performing a coculture experiment using PBMCs and HK-2 cells. We found that *RasGRP4* deletion could suppress the activation of the IL-17 signaling pathway and effector factors in HK-2 cells triggered by the interaction between M1 macrophages and CD4^+^ T cells.

Consistent with previous clinical studies (4, 27), our experimental results simultaneously demonstrate that diabetes plays a substantial role in the aggravation of kidney IRI. Inflammation and fibrosis, as the core of kidney disease progression, typically manifest in diabetic kidneys with reduced oxygenation, vascular damage, and enhanced inflammatory responses, which increase the susceptibility of the kidney to hypoxia (1). Concurrently, diabetes severely affects the kidneys’ self-repair capabilities and the communication within resident renal cells (28). Diabetic kidneys typically present with decreased oxygen supply, vascular injury, and intensified inflammatory reactions, thereby augmenting the kidneys’ vulnerability to hypoxic conditions (1). Consequently, the adverse effect of diabetes on the kidneys is inevitable. When concurrent with diabetes, hyperglycemia can serve as an ideal substrate that fosters a proinflammatory environment, exacerbating the inflammatory state of AKI. Hyperglycemia can induce the production of adhesion molecules and chemokines in renal proximal tubular cells, stimulating the expression of IL-8, ROS, TLRs, complement components, and complement receptors by tubular epithelial cells (29). These factors can lead to increased immune cell infiltration (such as macrophages and lymphocytes; refs. 30, 31) and renal injury. In this study, DI/R kidneys exhibited more pronounced renal functional impairment, infiltration of M1 macrophages and Th17 cells, activation of the IL-17 pathway, secretion of cytokines and effector molecules, and progression of tubular damage and renal fibrosis compared with NI/R kidneys. Although some results exhibited no apparent statistical differences, the role of diabetes in increasing susceptibility to renal IRI cannot be ignored. These effects were significantly mitigated after the deletion of *RasGRP4*, confirming its crucial role in diabetic IRI-damaged kidneys. Moreover, IL-17A inhibitor treatment not only suppressed the activation of the downstream IL-17 signaling pathway but also had a partial inhibitory effect on the expression of the M1 macrophage effector cytokines IL-6 and IL-1B, as well as the key Th17 transcription factors PSTAT3 and RORC, thereby rescuing kidney injury. These results reflect the Th17 immune response and that IL-17 signaling pathway serves an important role in diabetic IRI-damaged kidneys.

Previous studies revealed that ER stress, Klotho, and apoptosis appear to be involved in the pathogenesis of AKI-diabetes comorbidity (32, 33) as well as in increased proinflammatory pathways activated and neutrophil infiltration in the diabetic IRI model (34). According to Wu et al.’s research, SARS-CoV-2 N protein-induced AKI in db/db mice is associated with Mincle-dependent M1 macrophage activation (35). A COX-2/iPGE2/DPP-4 cascade was verified to also have a relevant role in the proximal tubular damage in septic patients with diabetes (36). In our study, we initially explored the role and mechanisms of *RasGRP4* in diabetic kidney IRI from the perspective of immune-inflammatory cellular responses, by investigating the effect of *RasGRP4* on the interactions between macrophages and CD4^+^ T cells, as well as the potential signaling pathways it may regulate. However, our study did not further validate the mechanisms in human samples, which will be the focus of our subsequent research efforts. An inflammatory storm may have been initiated by interactions among M1 macrophages, Th17 cells, and HK-2 cells, exacerbating the progression of DI/R. *RasGRP4* may thus constitute a potential therapeutic objective for the management of this state, and strict glycemic control in affected patients in clinical practice may help diminish the incidence and severity of AKI and enhance the prognosis of patients with AKI.

To further translate our discoveries into effective clinical treatment strategies, we propose the following options: (a) developmening small molecule inhibitors or antibodies that specifically target *RasGRP4* to modulate the inflammatory response; (b) exploring immunomodulatory therapies aimed at reducing the activation and infiltration of M1 macrophages and Th17 cells in the kidney, which may help in mitigating the inflammatory storm associated with DI/R; (c) developing educational programs for healthcare providers to increase awareness about the importance of glycemic control in preventing AKI and to disseminate knowledge about new treatment options; and (d) validating *RasGRP4* and other identified markers (e.g., IL-17, M1 macrophage markers) as predictive biomarkers for AKI in patients with diabetes, which could aid in early diagnosis and risk stratification.

In conclusion, the upregulation of the signaling protein *RasGRP4* under the condition of HH/R injury can induce the M1 polarization and activation of macrophages. This process subsequently facilitates the differentiation of CD4^+^ T cells into Th17 cells and enhances the expression of pivotal intracellular transcription factors, such as STAT3 and RORC. The secretion of IL-17A is thereby triggered, activating the IL-17 signaling pathway within renal tubular epithelial cells and leading to the associated effector factors release. Among them, the chemokines CCL2 and CCL3 are implicated in further macrophage recruitment, initiating a cascading inflammatory response that intensifies the renal inflammatory storm, which contributes to the initiation and progression of inflammatory injury in settings of renal IRI–related damage. DM aggravates renal IRI by amplifying these inflammatory mechanisms. The present study highlights *RasGRP4* as a prospective target for the prevention and treatment of DI/R.

## Methods

### Sex as a biological variable.

Our study was confined to male mice due to their less phenotypic variability. The applicability of these results to female mice remains to be determined.

### Animal model.

**Figure 13:**
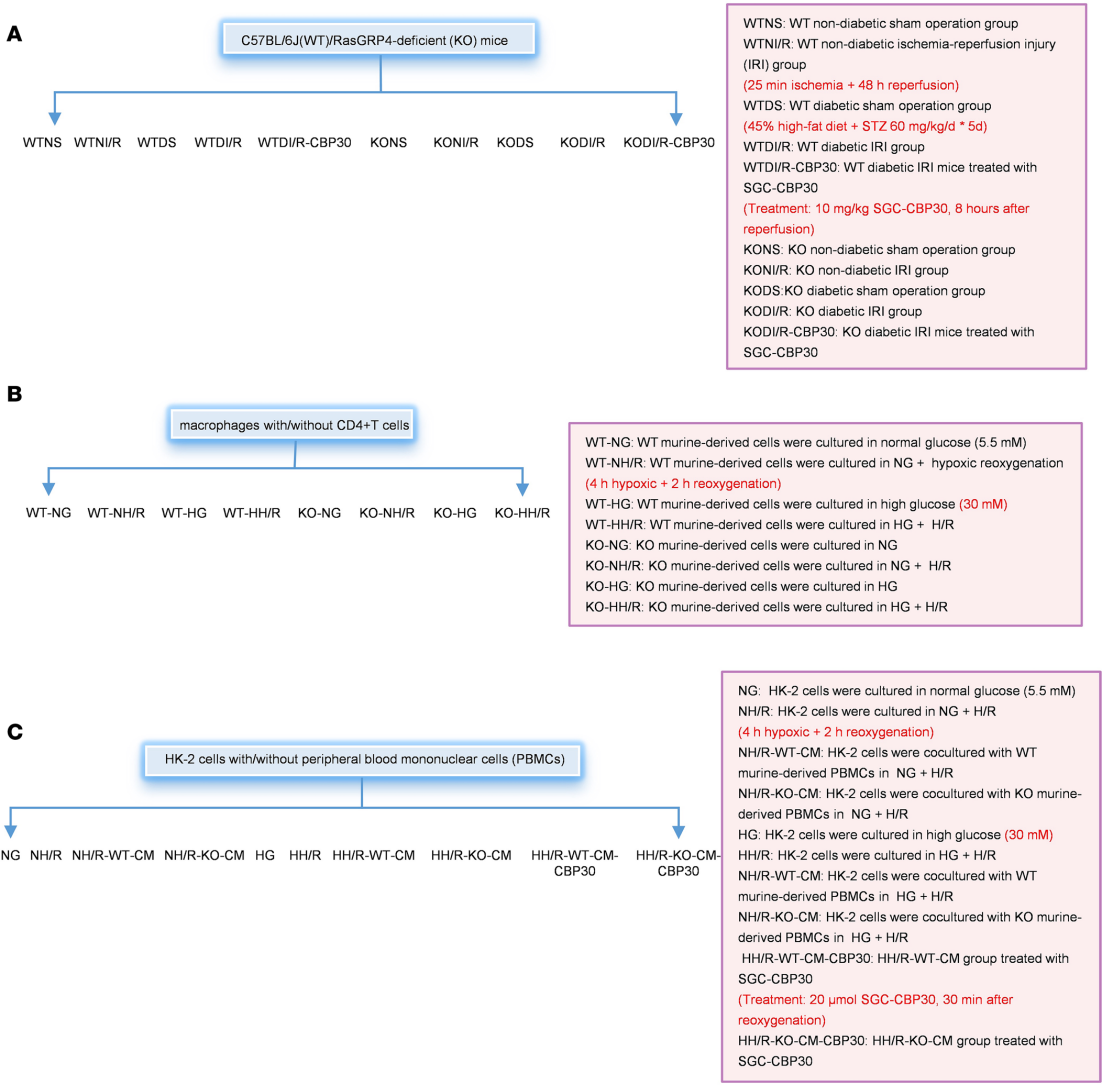
Schematic portrayal of the groups/treatment for both in vivo and in vitro investigations. (**A**) Experimental design and treatments for in vivo models of diabetic ischemia-reperfusion renal injury. (**B**) Cocultured design of Macrophage and CD4^+^ T cell. (**C**) Design and treatments for in vitro models of diabetic ischemia-reperfusion renal injury.

C57BL/6J male (WT) mice were sourced from GemPharmatech company. Mice with a *RasGRP4* gene deficiency (KO) on the C57BL/6J genetic background were generated through primary breeding, as described in our recent work (19). As illustrated in Figure 13A, both normal and diabetic mice were randomly allocated to different groups by a researcher not involved in the subsequent experimental procedures, ensuring that the operators were blinded to the group assignments.

Mice were maintained under specific pathogen–free (SPF) conditions with freely available water and standard mouse chow. Mice that were 6–8 weeks old with similar body weights were used to induce T2DM with an 8-week regimen of a 45% high-fat diet feeding coupled with 5-days i.p. injections of 60 mg/kg streptozotocin. A control group was administered an equivalent volume of 0.1 mmol/L sodium citrate buffer (Solarbio, C1013).

Following anesthesia induced via the i.p. injection, the skin was incised near the spinal column to separate the kidneys. In the renal IRI group, the bilateral renal pedicles of the mice were clamped with an arterial clamp for 25 minutes. The color change of the kidneys from bright red to purplish red was indicative of successful blocking, and a change from purplish red to bright red after loosening the arterial clamp was indicative of successful renal reperfusion. The reperfusion time was 48 hours, and animals in the drug-administration group were i.p. injected with 10 mg/kg SGC-CBP30 (TargetMol, T7573L) at 8 hours after reperfusion. In the sham operation group, all other surgical procedures were the same as those in the experimental groups, besides not occluded renal pedicles.

### Macrophage extraction.

Mice were administered 3% thioglycollate broth via i.p. injection for 3 consecutive days. On the fourth day, after sacrificed, PBS was injected into the mice abdominal cavity, stirred back and forth; all was aspirated out. After centrifugation (1,000 g), cells were resuspended with a complete culture medium and plated in different well plates. After a 4- to 6-hour interval, the culture medium was refreshed to remove nonadherent cells, leaving behind the adherent macrophages.

### Splenic CD4^+^ T cell sorting.

The mouse spleen was then isolated and processed through grinding and filtration to produce a single-cell suspension. This suspension was resuspended in an appropriate volume of cell sorting fluid and adjusted to a concentration of 1 × 10^8^ cells/mL in preparation for cell sorting. Sorting reagents A and B (Precision Biomedicals, 720410) were sequentially introduced to each 100 μL aliquot of the cell suspension. Following incubation at room temperature and exposure to a magnetic field, the supernatant, which contained the CD4^+^ T cells, was carefully collected after the magnetically labeled cells were retained.

### Macrophage and CD4^+^ T cell coculture.

After plating the collected macrophages, the medium was replaced with CD4^+^ T cells mixed with media of different sugar concentrations after 4–6 hours. After culturing for 48 hours, the hypoxia-reoxygenation group then had its media replaced with glucose-free (glucose-free 1640 [Pricella, PM150110] + 1% penicillin-streptomycin [Solarbio, P1400] + 0.1% mycoplasma inhibitor [Beyotime, C0288S]) complete culture medium and was cultured under hypoxic (37°C, 0.1% O_2_, 5% CO_2_) conditions for 4 hours, after which it was replaced with low-glucose medium and cultured under normoxic conditions (37°C, 5% CO_2_) for 2 hours. Anti-CD3e (5–10 μg/mL) (Precision Biomedicals, AM1003110) and anti-CD28 (2 μg/mL) (Precision Biomedicals, AM1028110) were added to culture media in each group to maintain the activity of CD4^+^ T cells.

Cocultured cells were divided into different groups as depicted in Figure 13B.

### Flow cytometry.

After the macrophage–CD4^+^ T cell coculture experiment, T cells were centrifuged (1,000 g) and resuspended in anti-CD4/IgG2b isotype control (Thermo Fisher Scientific, 00-5523-00) for surface staining, followed by incubation with the Foxp3 fixation/permeabilization working solution (Thermo Fisher Scientific, 00-5523-00), and finally with anti–IL-17A/IgG2a isotype control (Thermo Fisher Scientific, 17-7177-81) for nuclear staining. After the cells had been resuspended in the cell sorting solution, the proportion of Th17 cells within each group was quantified using flow cytometry, with the resultant data being analyzed in the FlowJo software.

### ELISAs.

After the macrophage and CD4^+^T cell coculture experiment, cellular supernatants were collected, and IL-17A concentrations were measured with a mouse IL-17A ELISA Kit (Fankew, F2756-A).

### Isolation of murine primary peripheral blood mononuclear cells (PBMCs).

Isolation of mouse primary PBMCs was performed using the Ficoll density gradient centrifugation method (1,000 g), and the extraction steps were described in our recent publication (19). The cell grouping was the same as that in the macrophage and CD4^+^ T cell coculture experiment.

### Multiplex immunoﬂuorescence staining.

After the routine dewaxing and heat restoration of renal paraffin sections, they were blocked and then subjected to incubation with primary anti-INOS (SAB, 53778)/anti-CD4 (Abmart, PU910880). The cells were then incubated with primary anti-F4/80 (Abmart, MU124812) and anti–IL-17A (Proteintech, 66148-1-Ig) at 4°C for 12 hours, followed by incubation with fluorescent secondary antibodies and nuclear staining with DAPI. Slides were mounted for fluorescence microscopy.

For PBMCs, they were fixed with 4% paraformaldehyde and permeabilized with 0.5% Triton. The subsequent blocking and antibody incubation steps were the same as above.

### Cell treatment.

Human Kidney-2 (HK-2) cells, obtained from the American Type Culture Collection (ATCC), were maintained in 5.5 mmol/L glucose media. The procedures for hypoxia-reoxygenation modeling were the same as above. Among them, in the drug-treated group, 20 μmol of SGC-CBP30 was added at 30 minutes of reoxygenation. Cells (cultured with/without PBMCs) were divided into different groups, as shown in Figure 13C.

### Biochemical measurements and histologic evaluation.

Liver and kidney function parameters, as well as lipid profiles, were assessed utilizing commercial assay kits, as detailed in our previous publications (19). Renal histology was examined by H&E staining, periodic acid–Schiff’s staining, and Masson staining.

### IHC.

After routine dewaxing and heat-mediated antigen repair, paraffin-embedded kidney sections were subjected to blocking. Sections were then exposed to specific antibodies for INOS (SAB, 53778), CD4 (Abmart, PU910880), F4/80 (Abmart, MU124812), and IL-17A (Proteintech, 66148-1-Ig) overnight at 4°C, followed by incubation with the respective secondary antibodies for 1 hour at 37°C. Following staining with diaminobenzidine and subsequent counterstaining with hematoxylin, the slides were examined using a microscope.

### Western blotting.

Proteins were resolved by sodium dodecyl sulfate-polyacrylamide gel electrophoresis and transferred to a nitrocellulose membrane. The membranes were blocked and incubated with primary antibodies at 4°C overnight (Biorbyt Ltd.: *RasGRP4,* S40999; Abmart: ECA, TA0131; FIBRONECTIN, T59537; GAPDH, P60037; PP65, TP56372; and P65, T55034; Proteintech: SMA, 67735-1-Ig; IL-17A, 66148-1-Ig; and PSTAT3, 28945-1-AP; ABclonal: AKLOTHO, A12028; PP38, AP1311; P38, A4771; IL-6, A11115; MMP3, A11418; IL-17RA, A10052; TNFA, A11534; RORC, A10240; and IL-1B, A1112; Beyotime: TGF-β, AF0297; Affinity: PERK, AF1015; Cell Signaling Technology: ERK, 4370; PTMbio: COX2, PTM-5167; SAB: EDN1, 41562; CCL2, 29548; CCL3, 30966; and INOS, 53778. The next day, HRP-conjugated secondary antibodies were applied and immunoreactive bands were detected. All immunoblotting assays were performed using 5 independent samples.

### qPCR.

Total RNA was extracted using the SPAR Keasy Improved Tissue/Cell RNA Kit (Sparkjade). Quantitative PCR (qPCR) was conducted (19), employing primers specific for mouse *RasGRP4*: forward 5′-GAGGGCGGGGTCGGTCACG-3′, reverse 5′-CGATCAAAGCAGCGGATACATTC-3′; mouse *IL1B*: forward 5′-CACTACAGGCTCCGAGATGAACAAC-3′, reverse 5′-TGTCGTTGCTTGGTTCTCCTTGTAC-3′; mouse *INOS*: forward 5′-ATCTTGGAGCGAGTTGTGGATTGTC-3′,reverse 5′-TAGGTGAGGGCTTGGCTGAGTG-3′; and mouse *IL6*: forward 5′-CTTCTTGGGACTGATGCTGGTGAC-3′, reverse 5′-TCTGTTGGGAGTGGTATCCTCTGTG-3′.

### Statistics.

Statistical analyses were performed using the GraphPad Prism software (v 9.0). Data were expressed as means ± SD. For comparisons between 2 groups, the 2-tailed independent-sample *t* test was utilized, whereas 1-way ANOVA was employed for assessing differences among multiple groups. A *P* value of less than 0.05 was considered to indicate statistical significance. Mice that succumb naturally during the modeling process of diabetes or renal ischemia-reperfusion were excluded.

### Study approval.

The animal experiments were reviewed and approved by the Ethics Committee of Chu Hsien-I Memorial Hospital of Tianjin Medical University (approval no. DXBYY-IACUC-2022083). All animal experiments were comply with Animal Research: Reporting of In Vivo Experiments (ARRIVE) guidelines.

### Data availability.

All data values depicted in the table and graphs are provided in the Supporting Data Values file.

## Author contributions

LZ contributed conceptualization, methodology, validation, investigation, data curation, writing of the original draft, and visualization. Zhanglong Wang contributed methodology, validation, investigation, data curation, and review and editing of the manuscript. YW contributed methodology, validation, investigation, data curation, and review and editing of the manuscript. BZ contributed methodology, validation, investigation, and data curation. Zhongli Wang contributed methodology, validation, investigation, and data curation. SC contributed funding acquisition and data curation. XM contributed funding acquisition and data curation. PY contributed supervision, project administration, and review and editing of the manuscript. SZ contributed supervision, project administration, funding acquisition, and review and editing of the manuscript.

## Supplementary Material

Supplemental data

Unedited blot and gel images

Supporting data values

## Figures and Tables

**Table 1:**
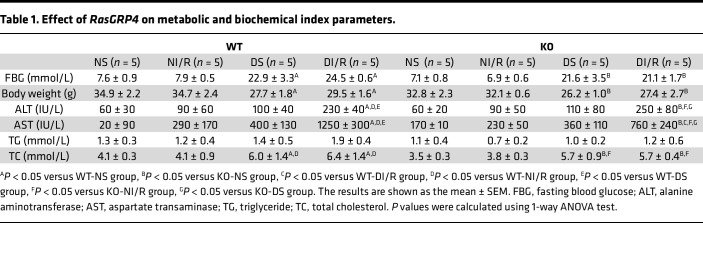
Effect of *RasGRP4* on metabolic and biochemical index parameters.

## Version 1 12/10/2024

In-Press Preview

## Version 2 01/23/2025

Electronic publication
