# Supplementary material for: RasGRP4 aggravates ischemia-reperfusion injury in diabetic kidneys by mediating communication between macrophages and T cells
Source: JCI Insight. 2024 Dec 10;10(2):e187653. doi: 10.1172/jci.insight.187653 (PMC11790033; doi:10.1172/jci.insight.187653)
Supplement: Supplemental data [file jciinsight-10-187653-s134.pdf]

Supplemental data

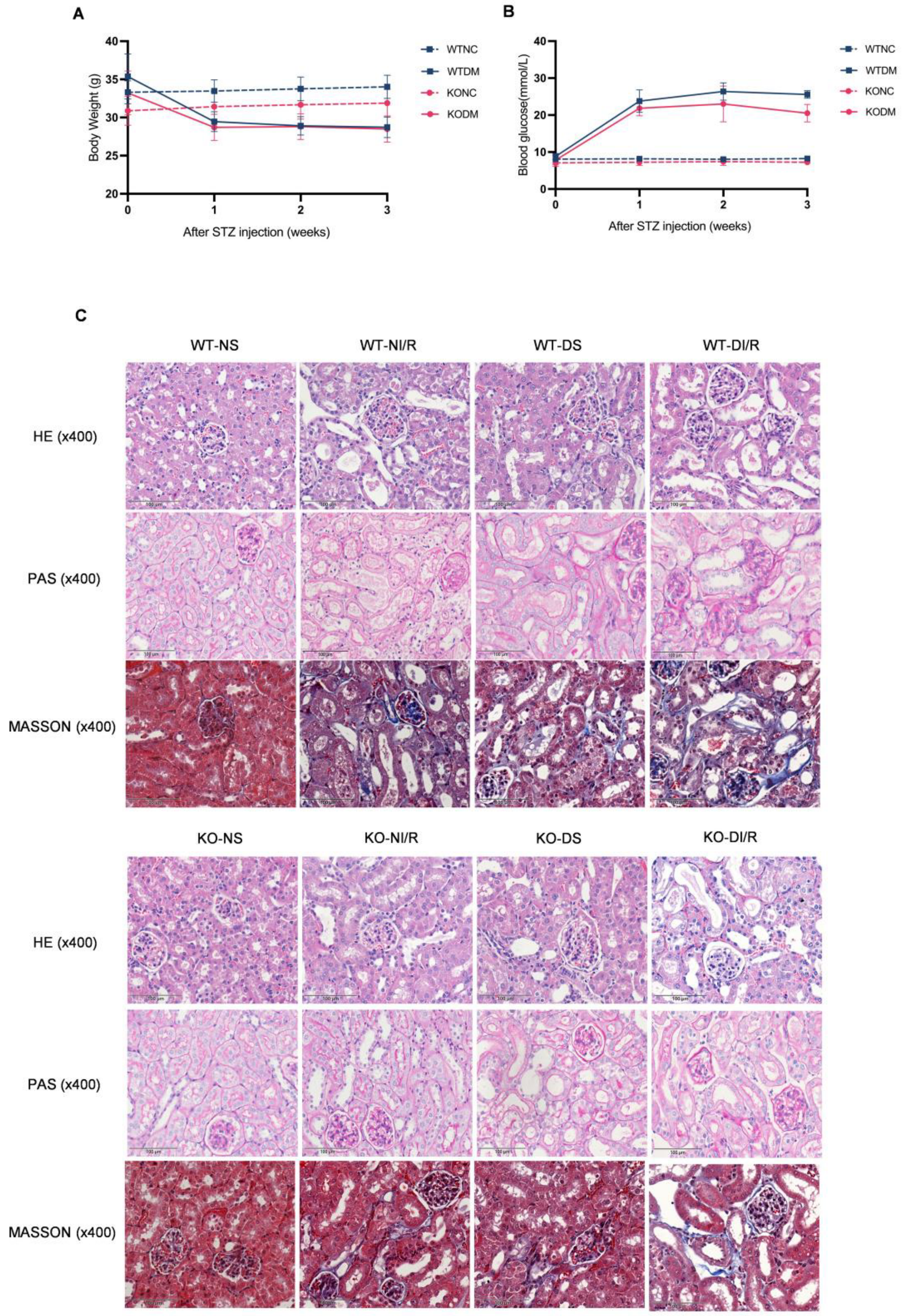

Supplemental Figure 1. RasGRP4 did not affect body weight or blood glucose levels but exacerbated renal pathological injury diabetic ischemia-reperfusion injury kidneys. A: Body weight (n = 10/group); B: blood glucose (n = 10/group); C: HE, PAS and

Masson staining of renal tissues, Scale bars: 100 μm.

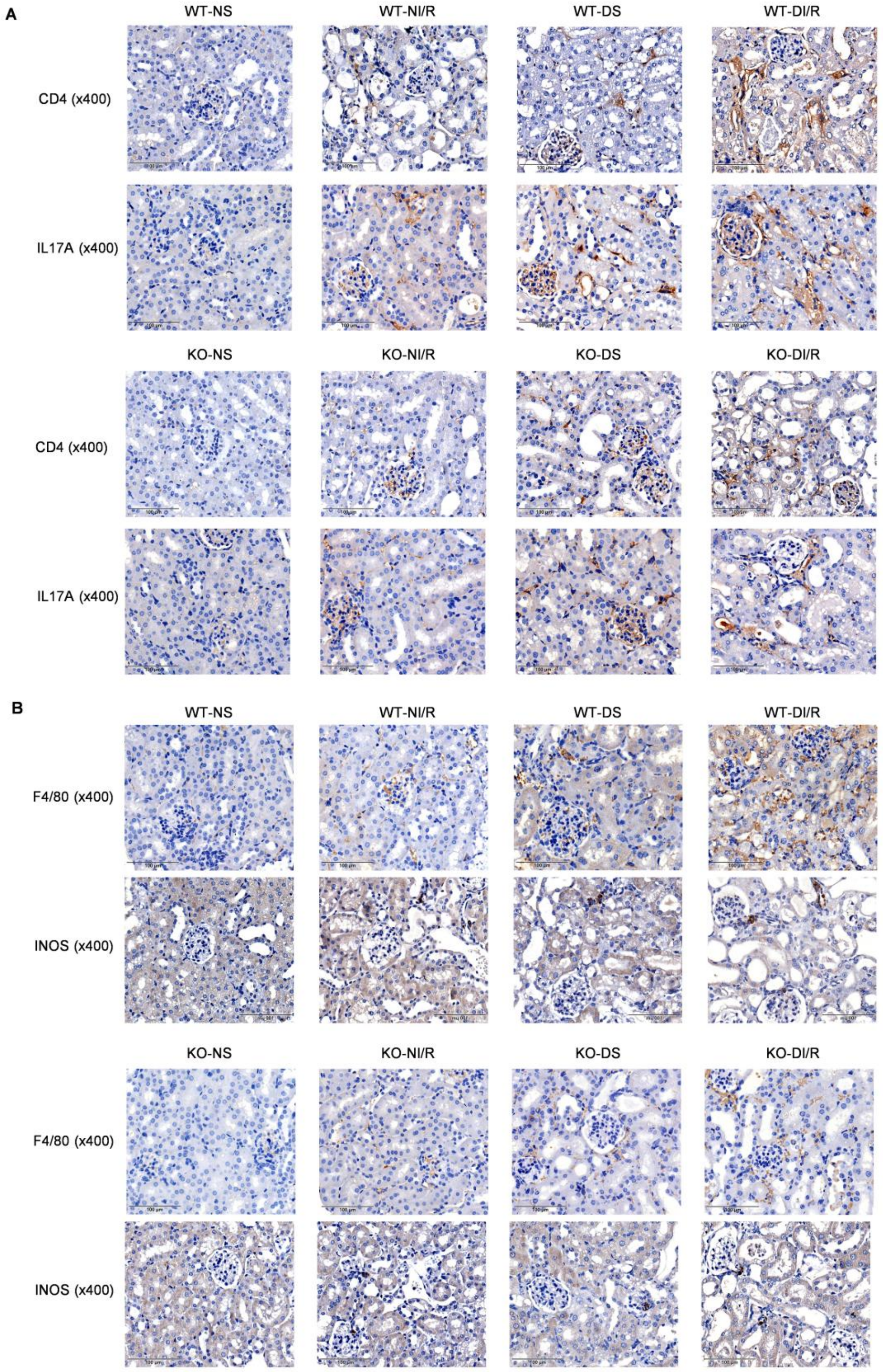

Supplemental Figure 2. RasGRP4 promoted infiltration of M1 macrophages and Th17 cells in the kidneys of diabetic ischemia-reperfusion injury.

A: Immunohistochemical staining of CD4 and IL17 in renal, Scale bars: 100 μm; B: Immunohistochemical staining of F4/80 and INOS in kidneys, Scale bars: 100 μm.

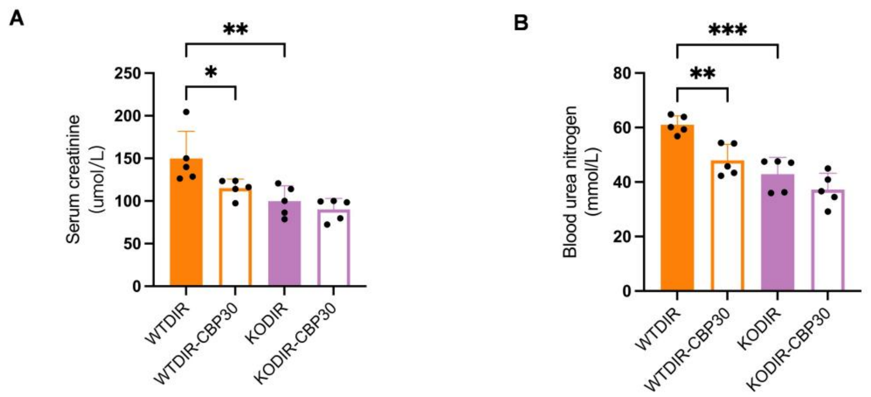

Supplemental Figure 3. IL17A inhibitor partially alleviated renal impairment.

A: serum creatinine (n = 5/group); B: blood urea nitrogen (n = 5/group).

*P < 0.05, **P < 0.01, ***P < 0.001

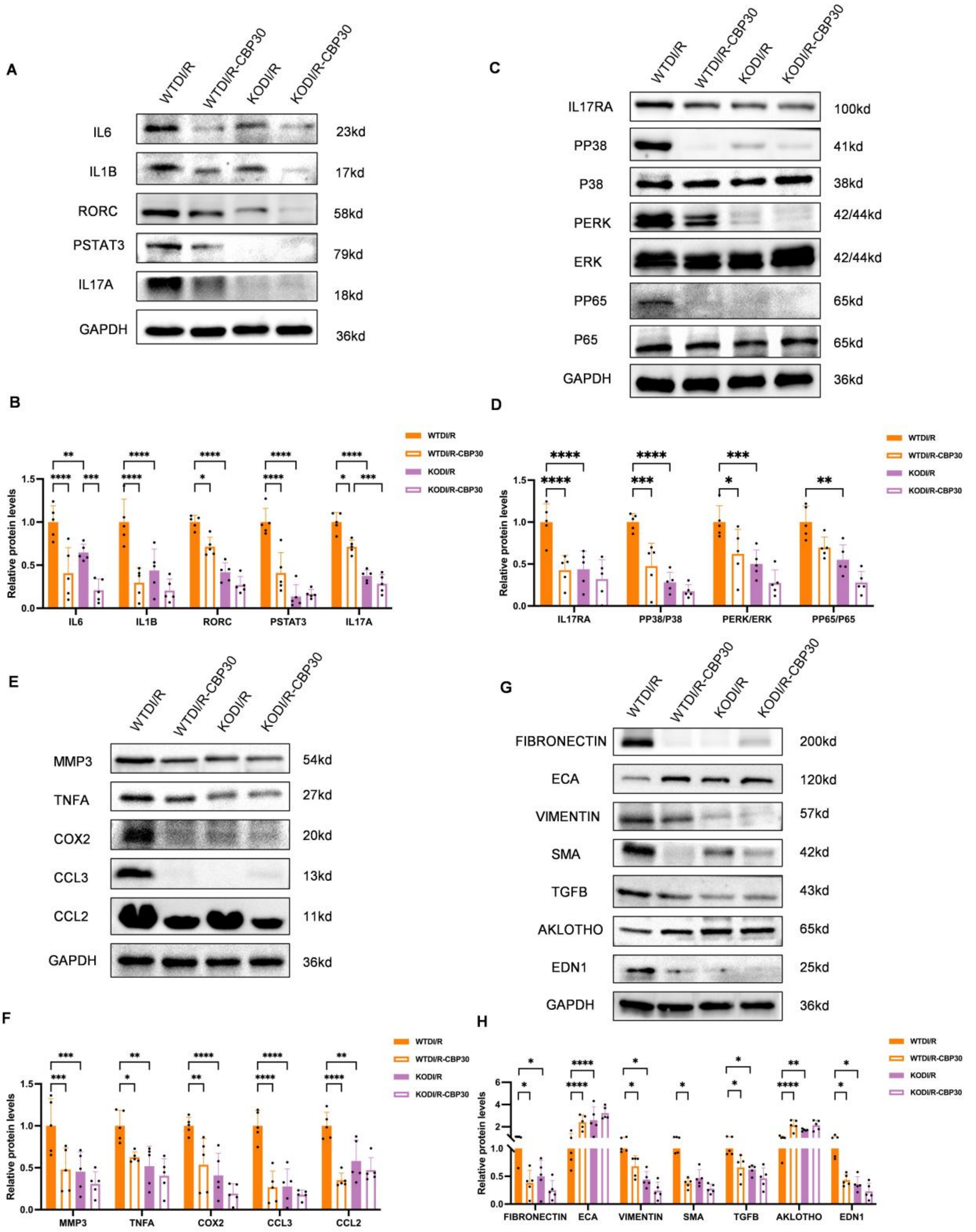

Supplemental Figure 4. IL17A inhibitor attenuated Th17 immune response and kidney inflammatory injury to a certain extent.

A-B: The protein expression levels and quantitative analyses of IL6, IL1B, RORC, PSTAT3, and IL17 in renal (n = 5/group); C-D: The protein expression levels and quantitative analyses of IL17RA, PP38/P38, PERK/ERK, and PP65/P65 in renal (n =

5/group); E-F: The protein expression levels and quantitative analyses of MMP3, TNFA, COX2, CCL3, and CCL2 in renal (n = 5/group); G-H: The protein expression levels and quantitative analyses of ECA, VIMENTIN, SMA,TGFB, AKLOTHO and EDN1 in renal (n = 5/group).

*P < 0.05, **P < 0.01, ***P < 0.001, ****P < 0.0001
