# Supplementary material for: RasGRP4 aggravates ischemia-reperfusion injury in diabetic kidneys by mediating communication between macrophages and T cells
Source: JCI Insight. 2024 Dec 10;10(2):e187653. doi: 10.1172/jci.insight.187653 (PMC11790033; doi:10.1172/jci.insight.187653)

Full unedited gel for Figure 1B

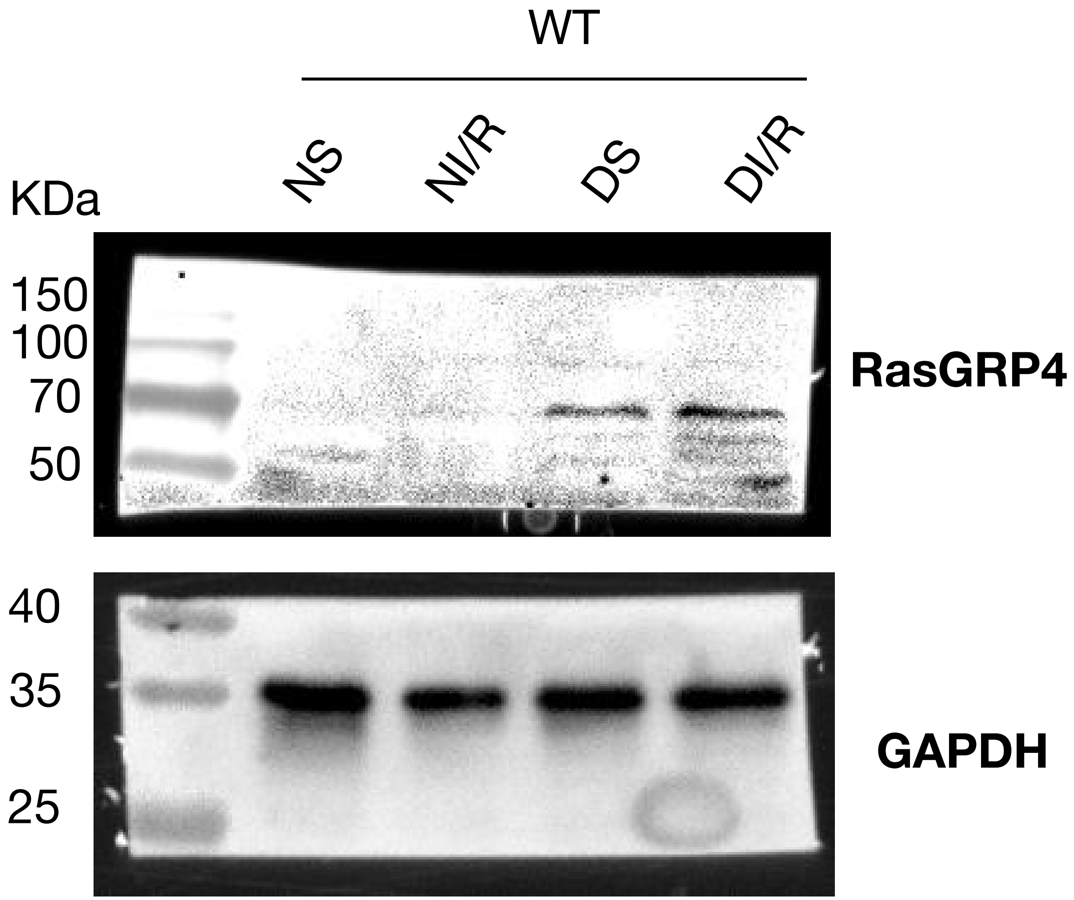

Full unedited gel for Figure 2A and 2F

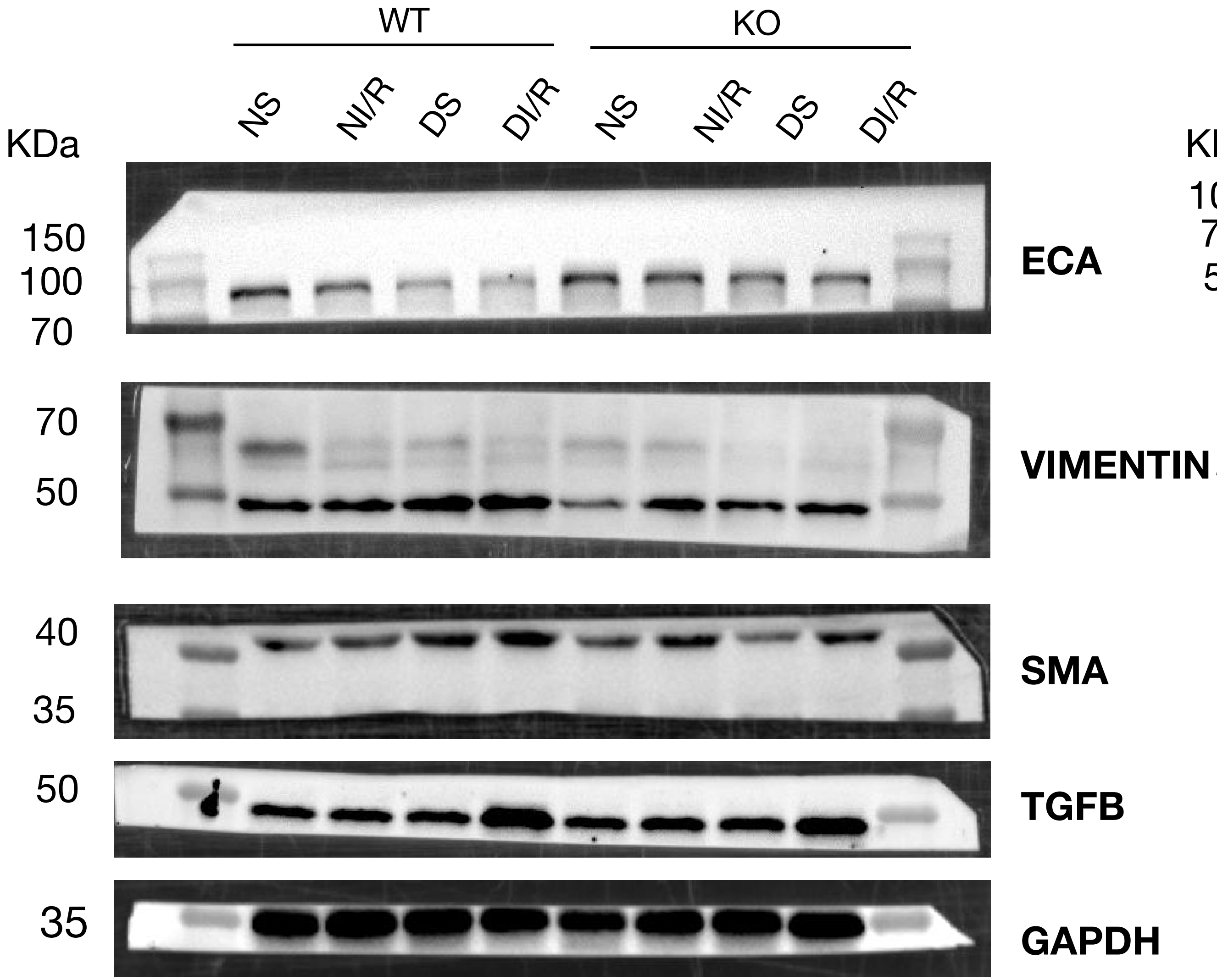

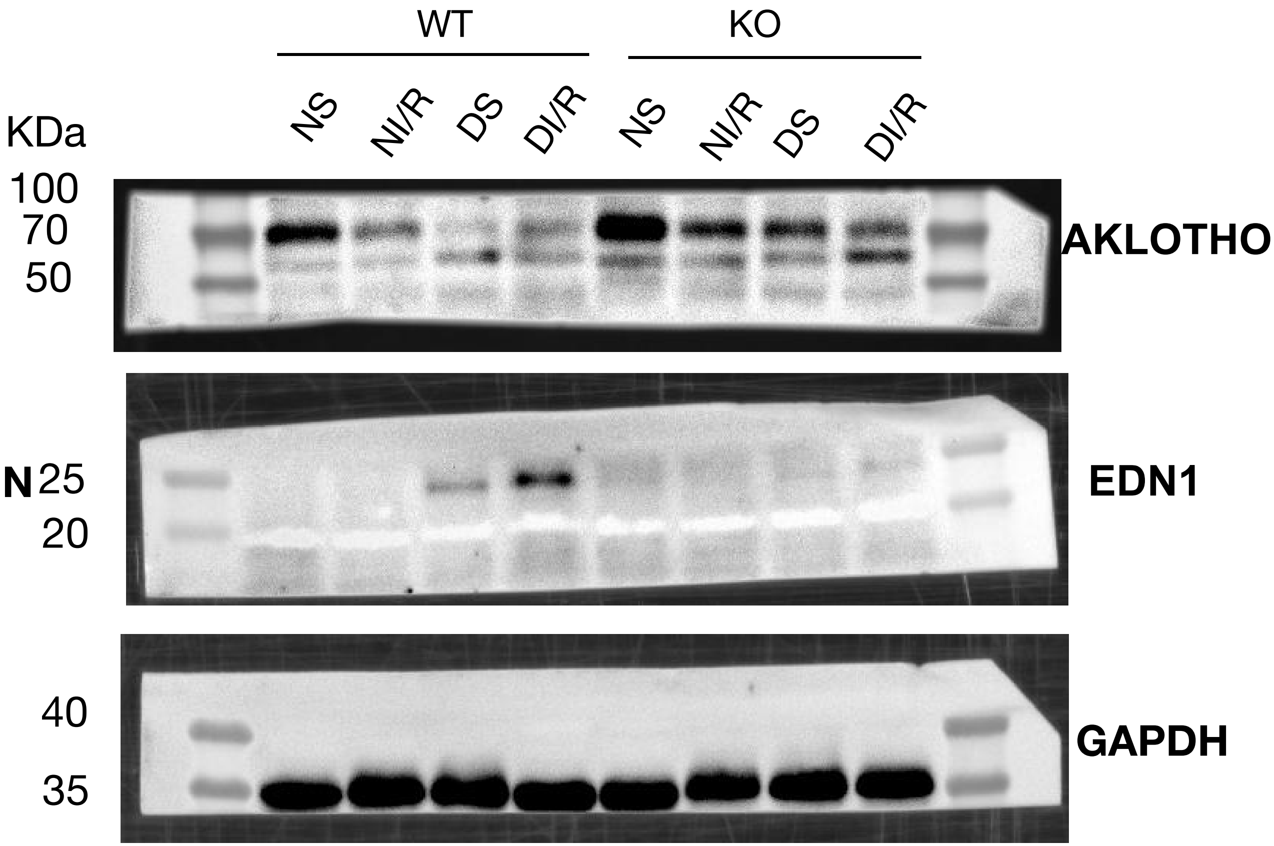

Full unedited gel for Figure 3C

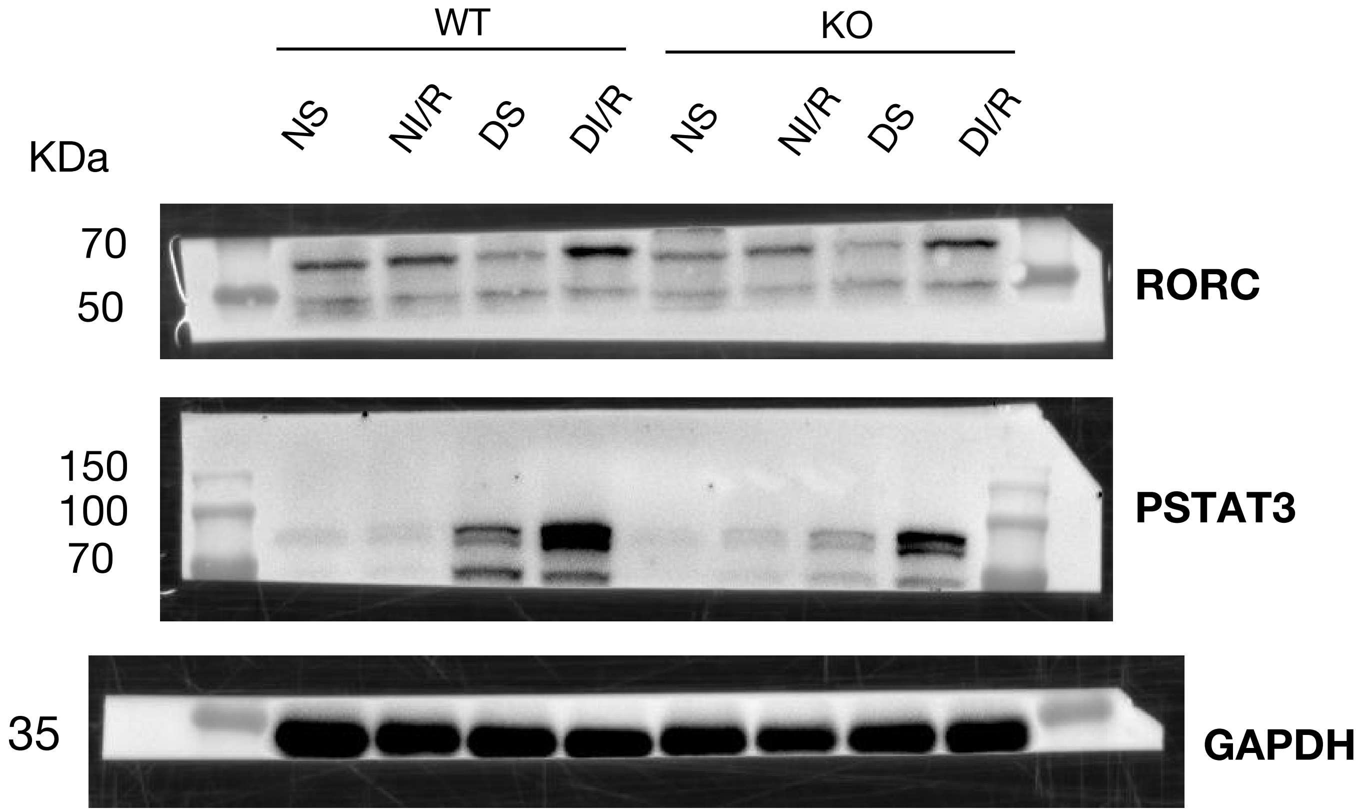

Full unedited gel for Figure 6D

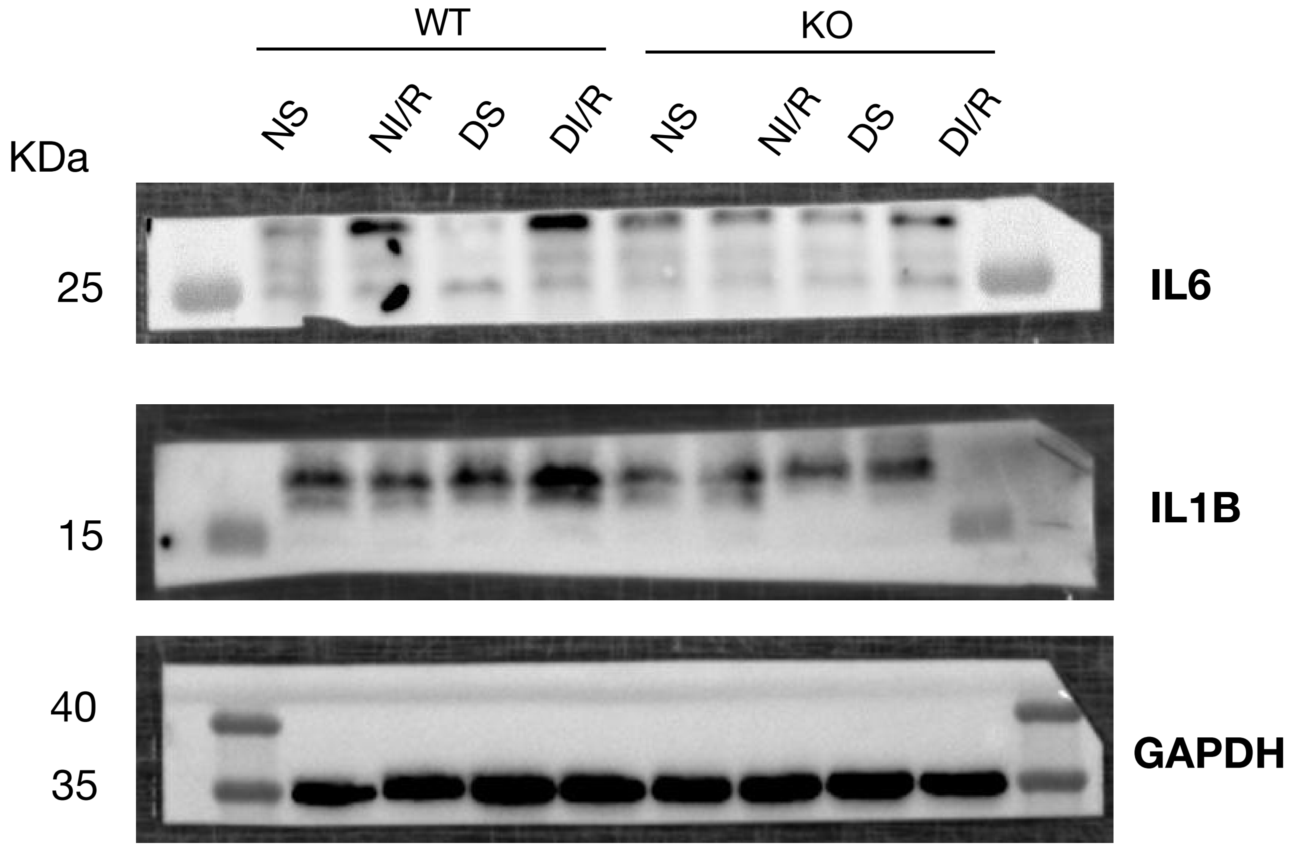

Full unedited gel for Figure 7A

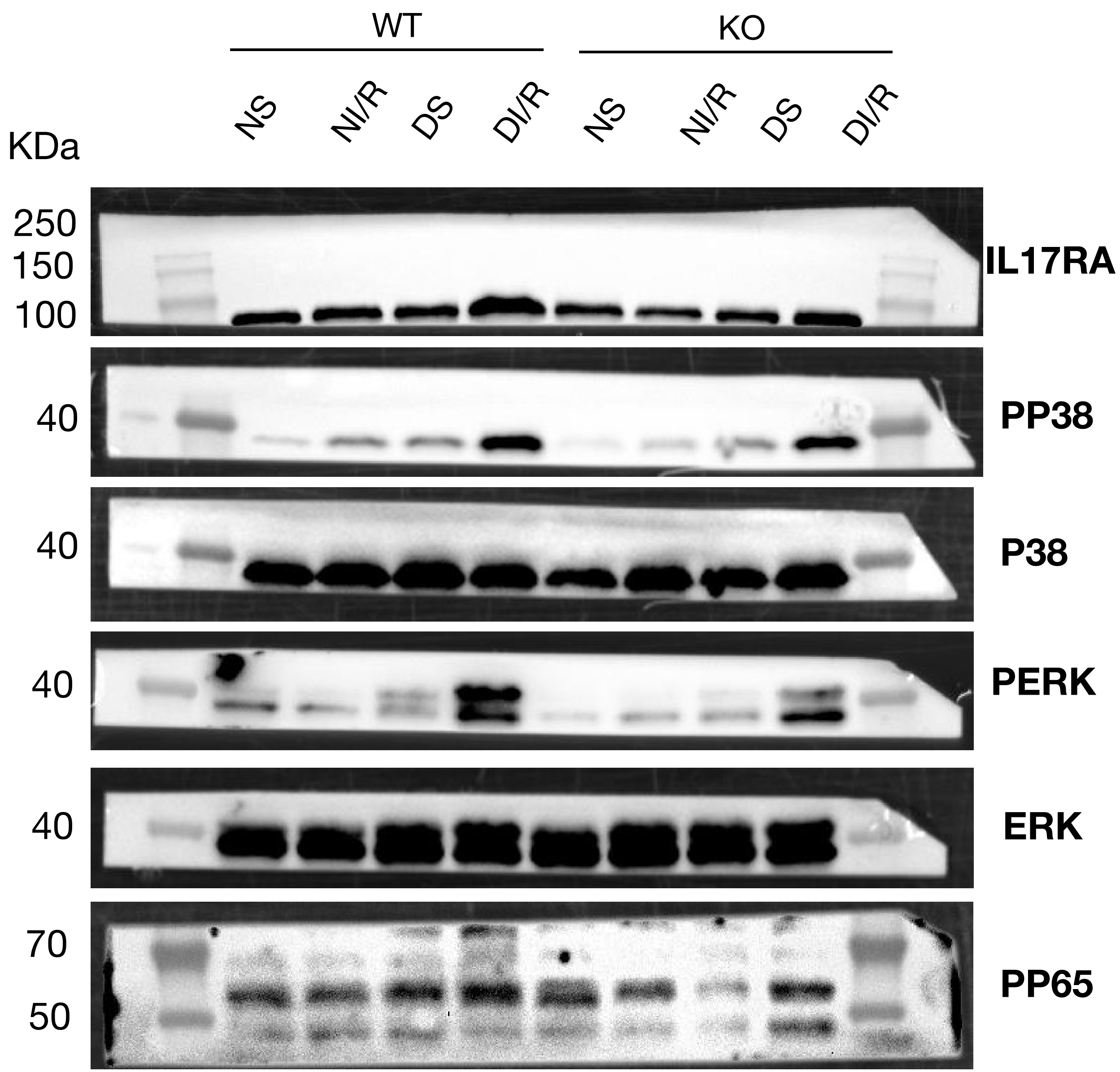

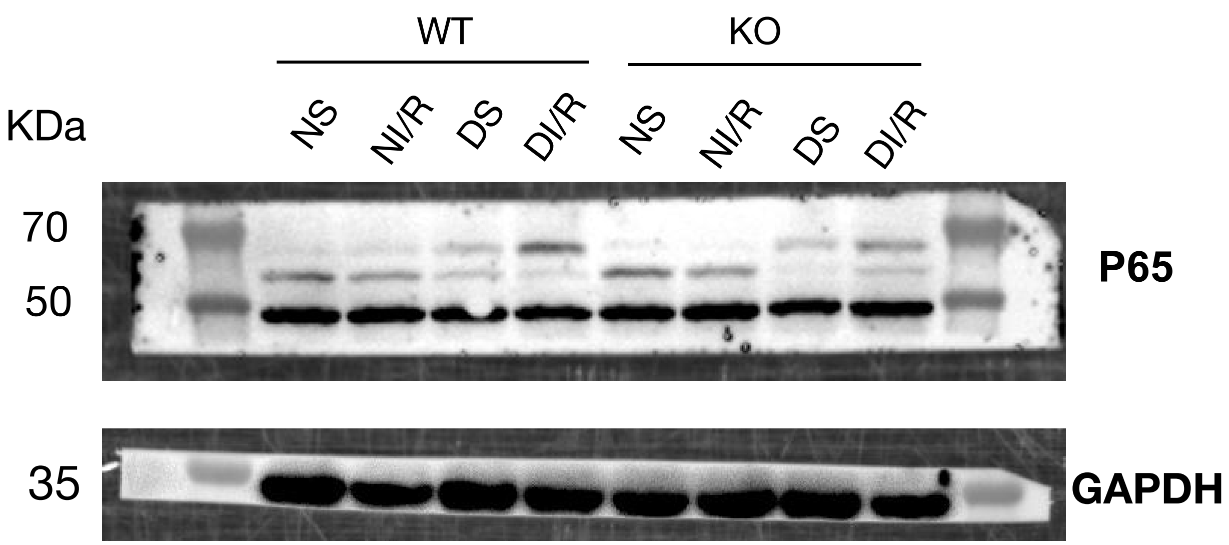

Full unedited gel for Figure 7F

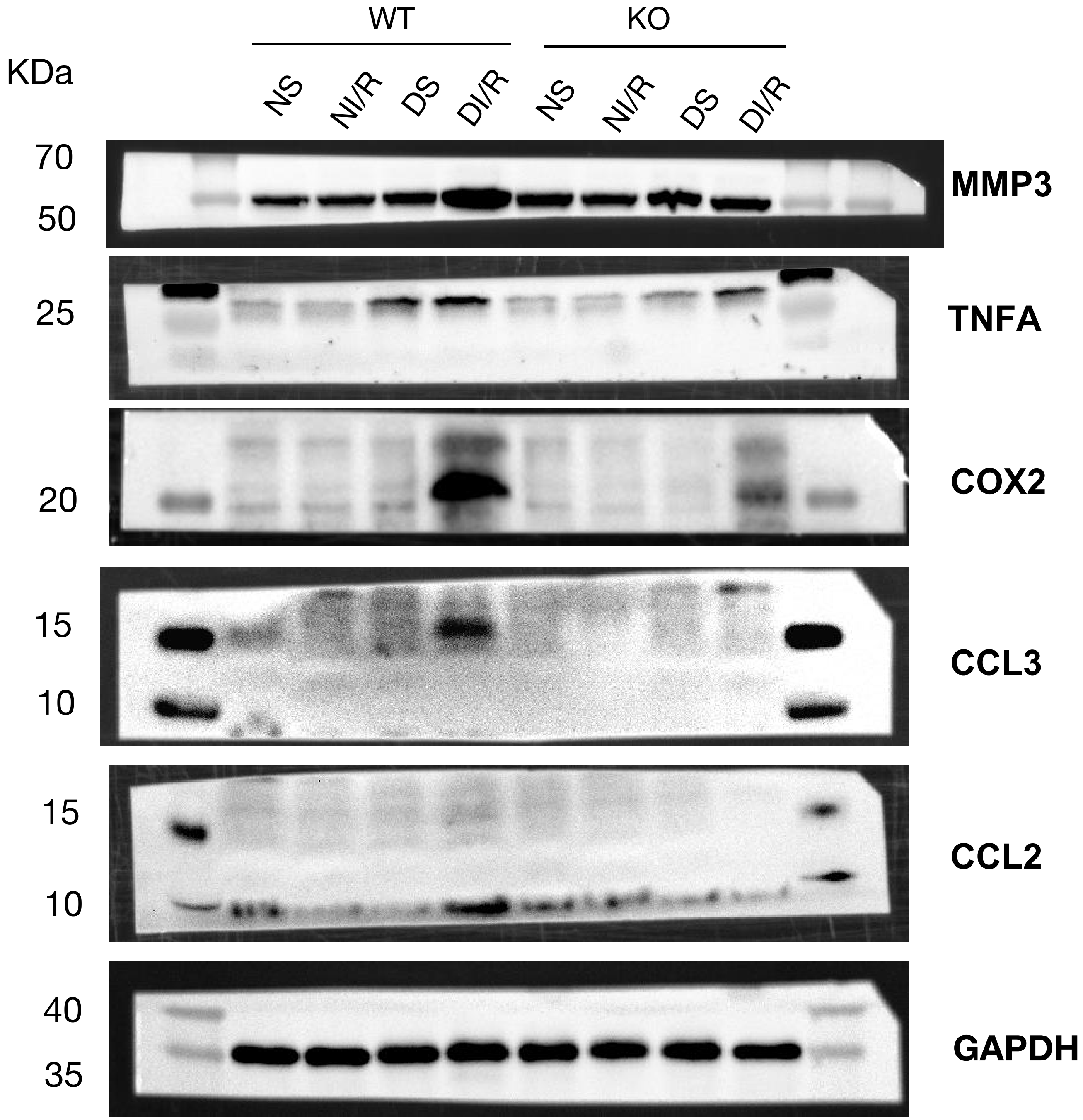

Full unedited gel for Figure 8A and 8F

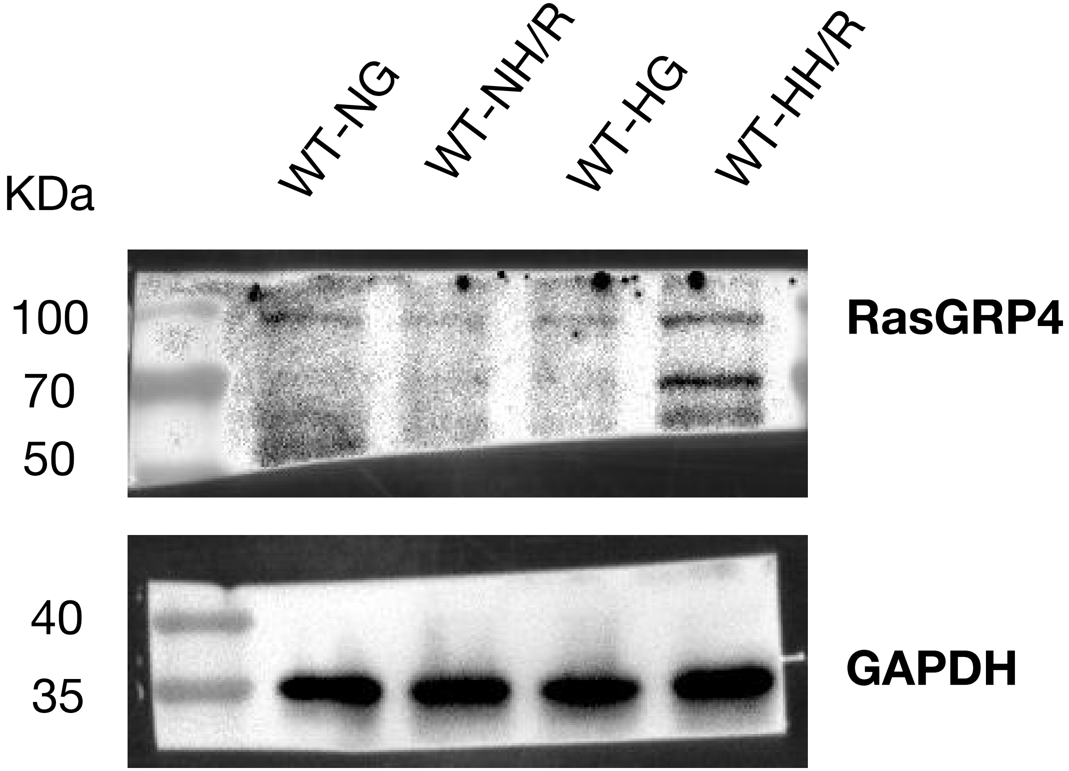

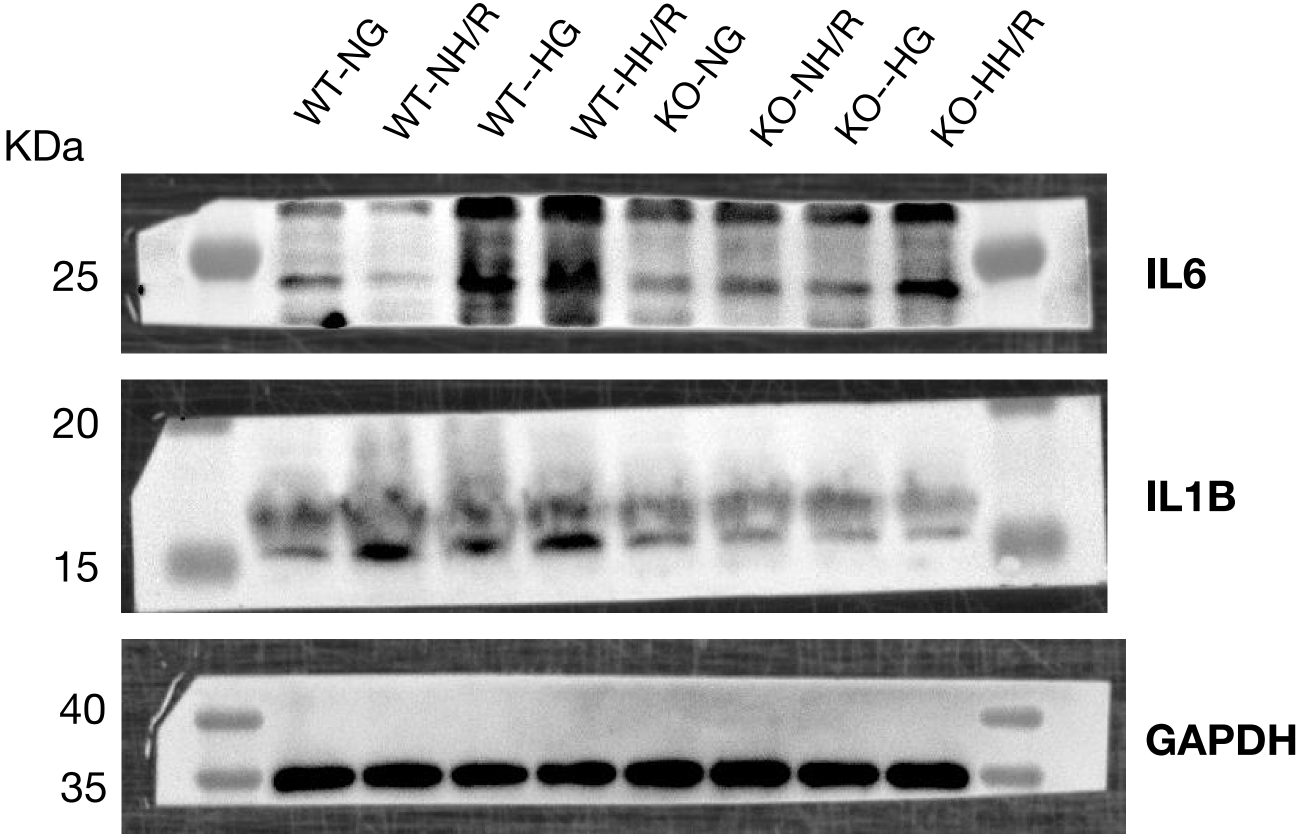

Full unedited gel for Figure 10A

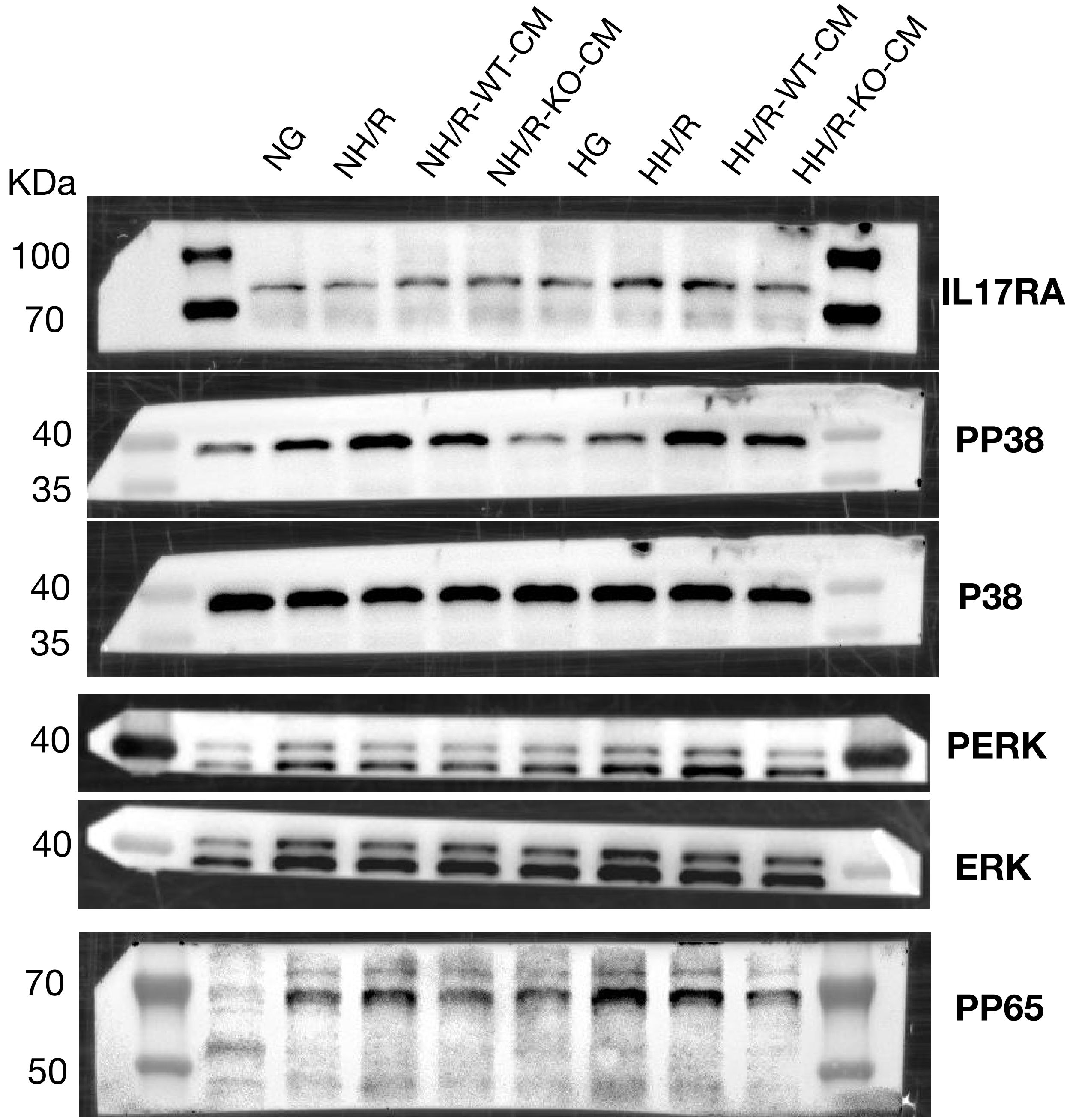

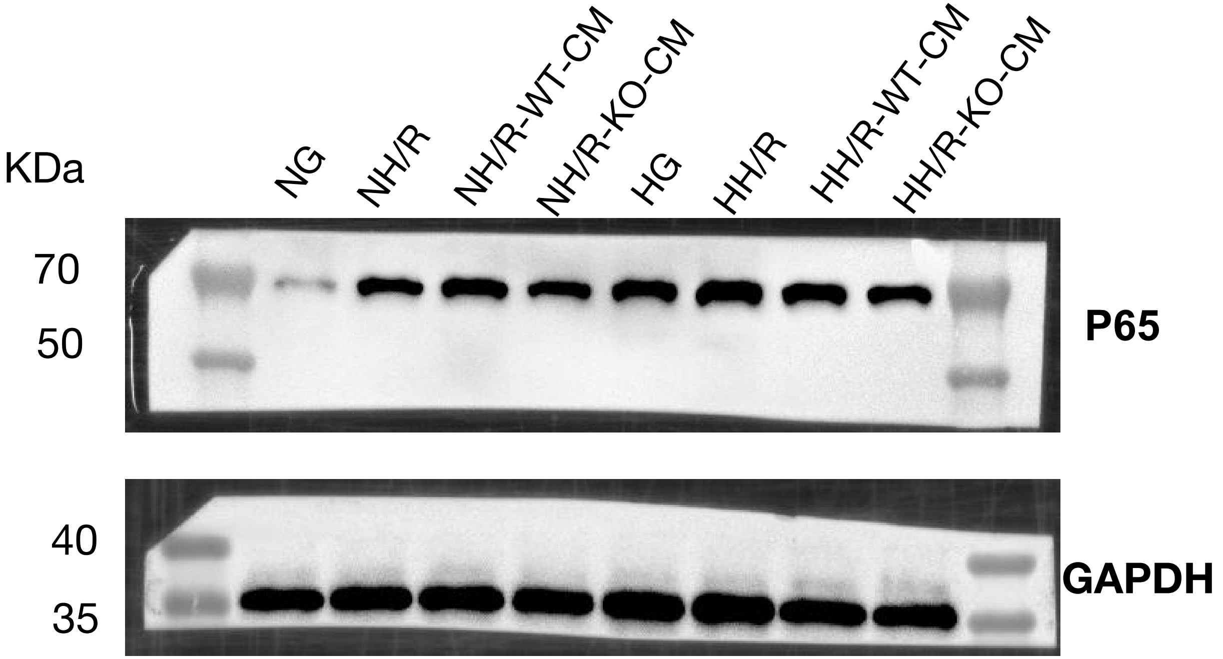

Full unedited gel for Figure 10F

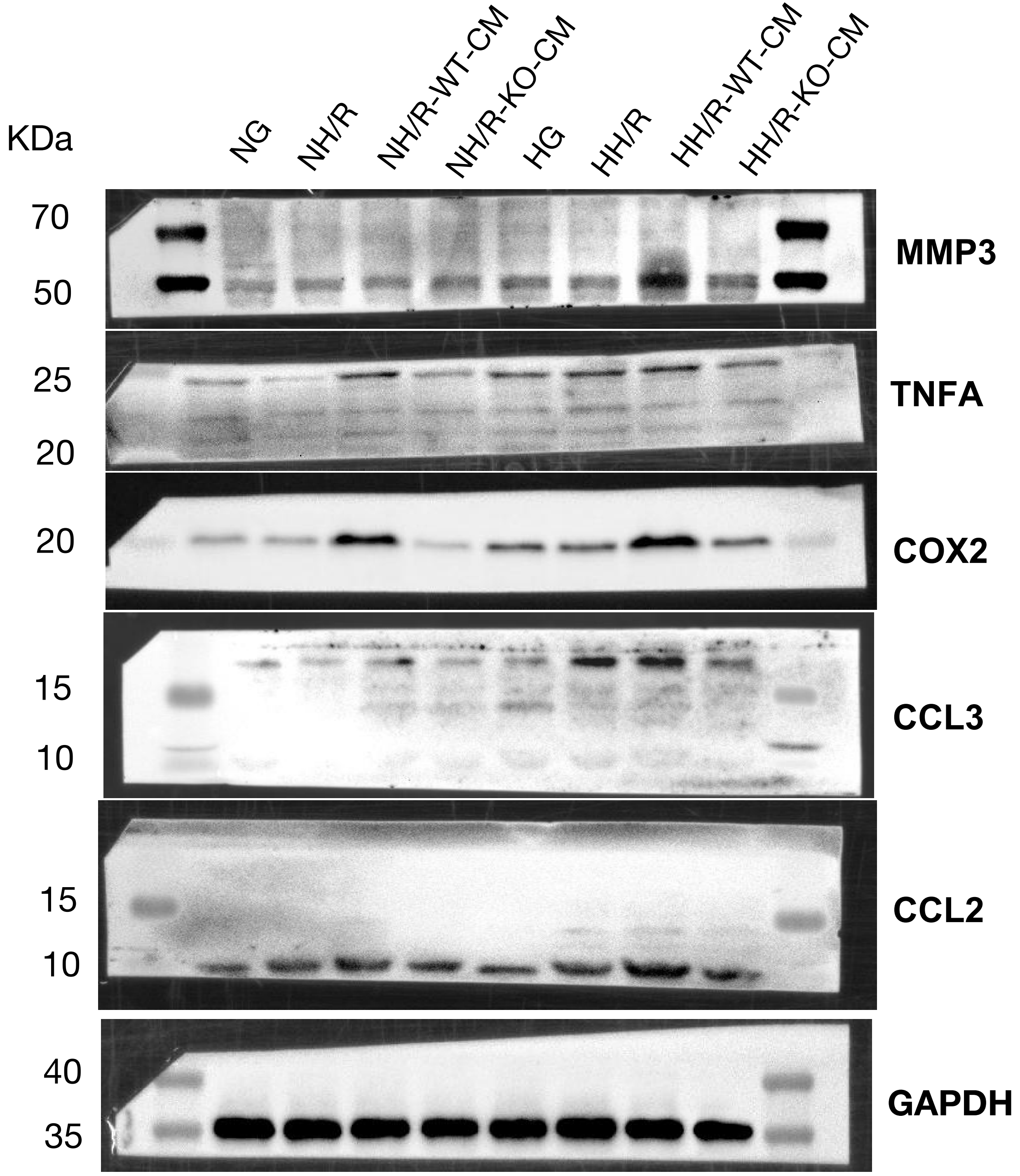

Full unedited gel for Figure 11A and 11G

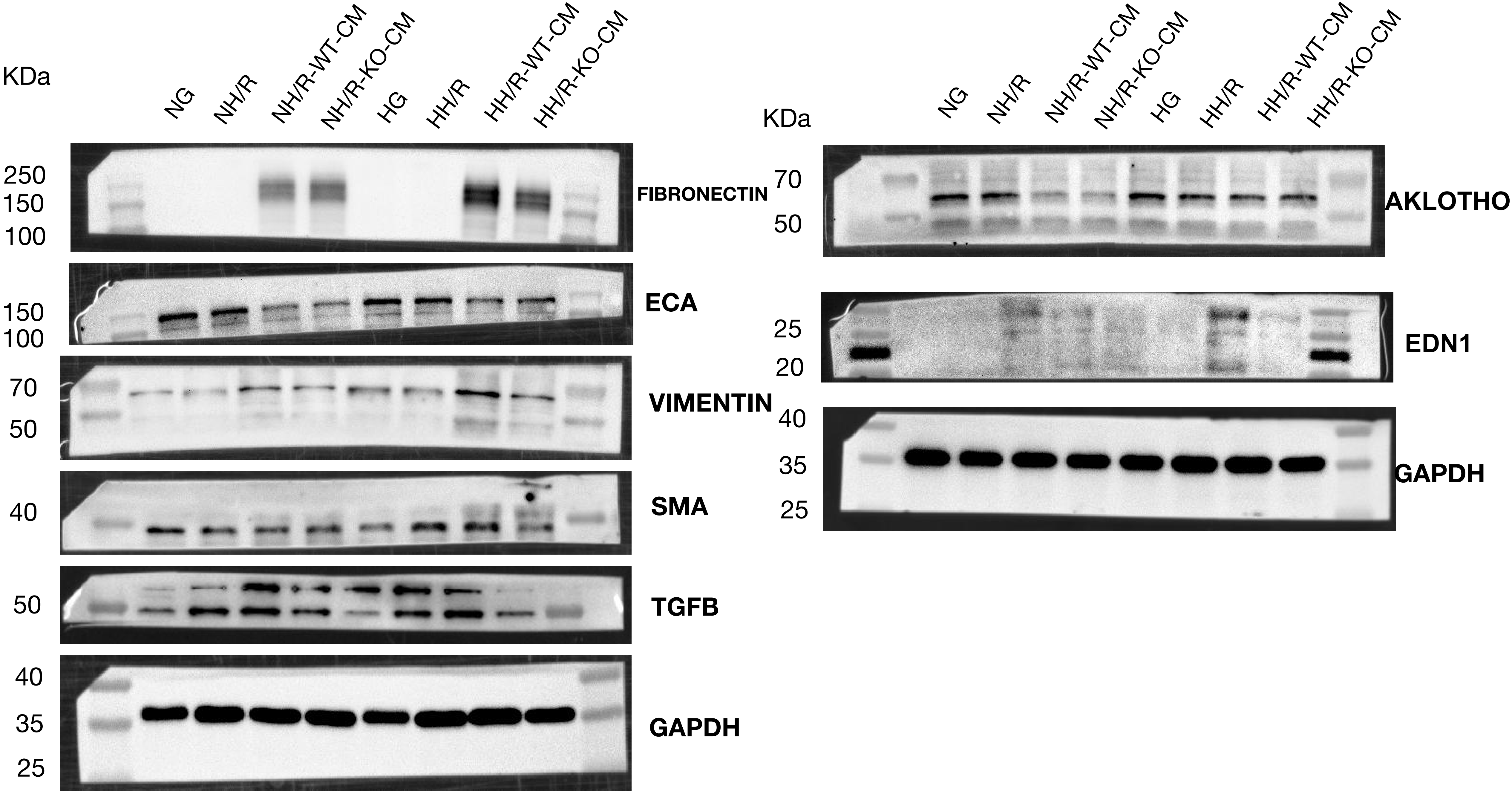

Full unedited gel for Figure 12A

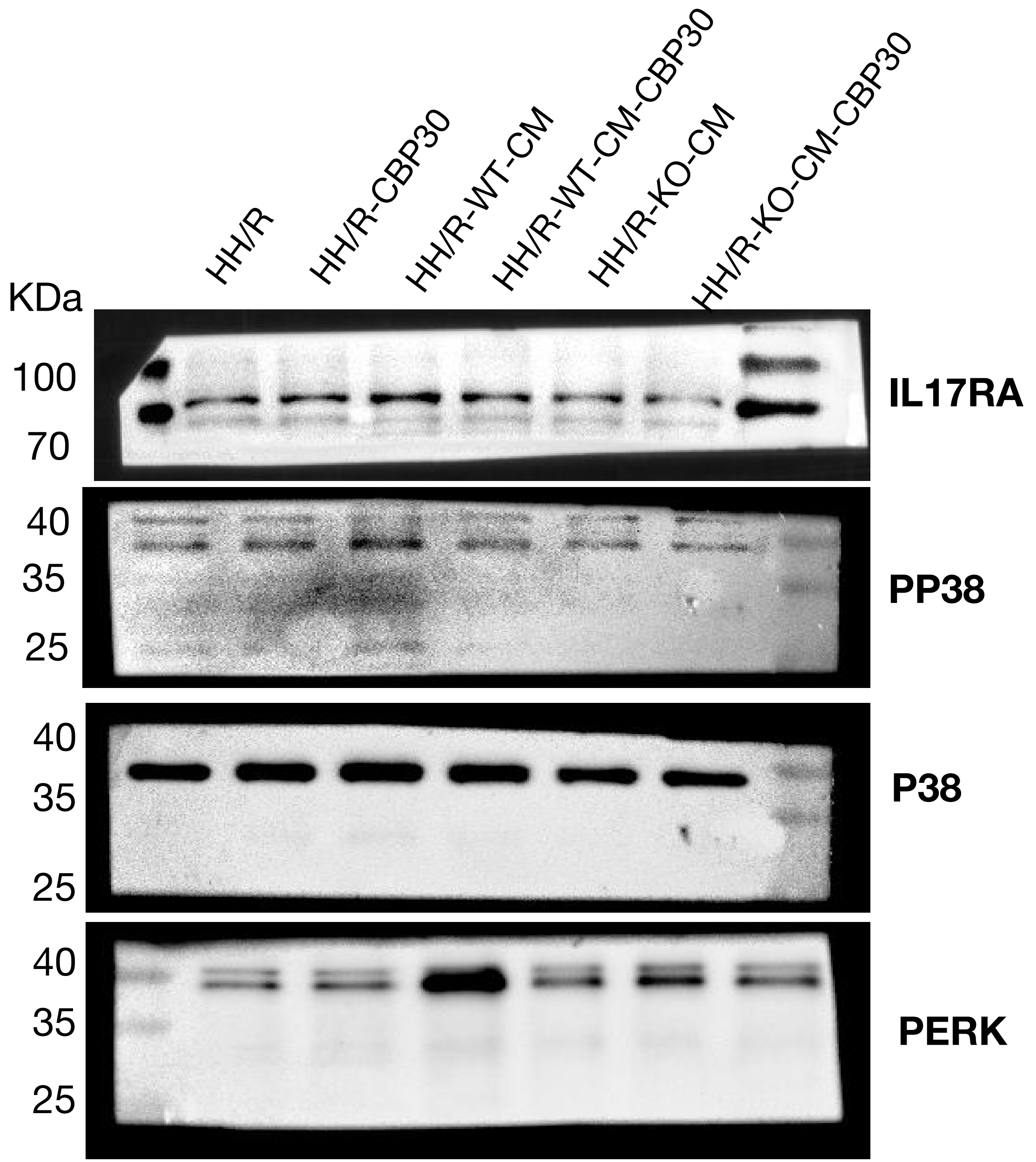

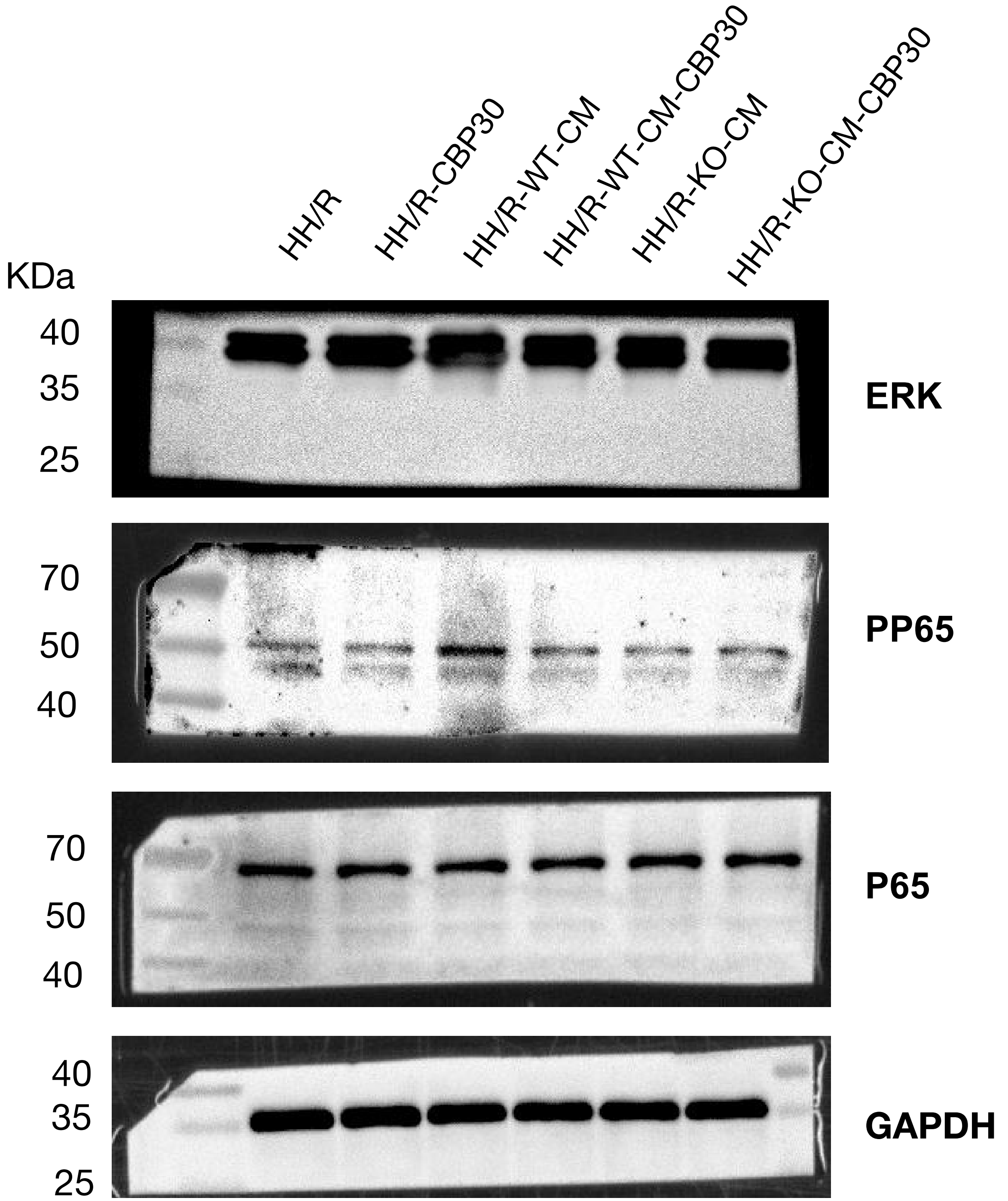

Full unedited gel for Figure 12F

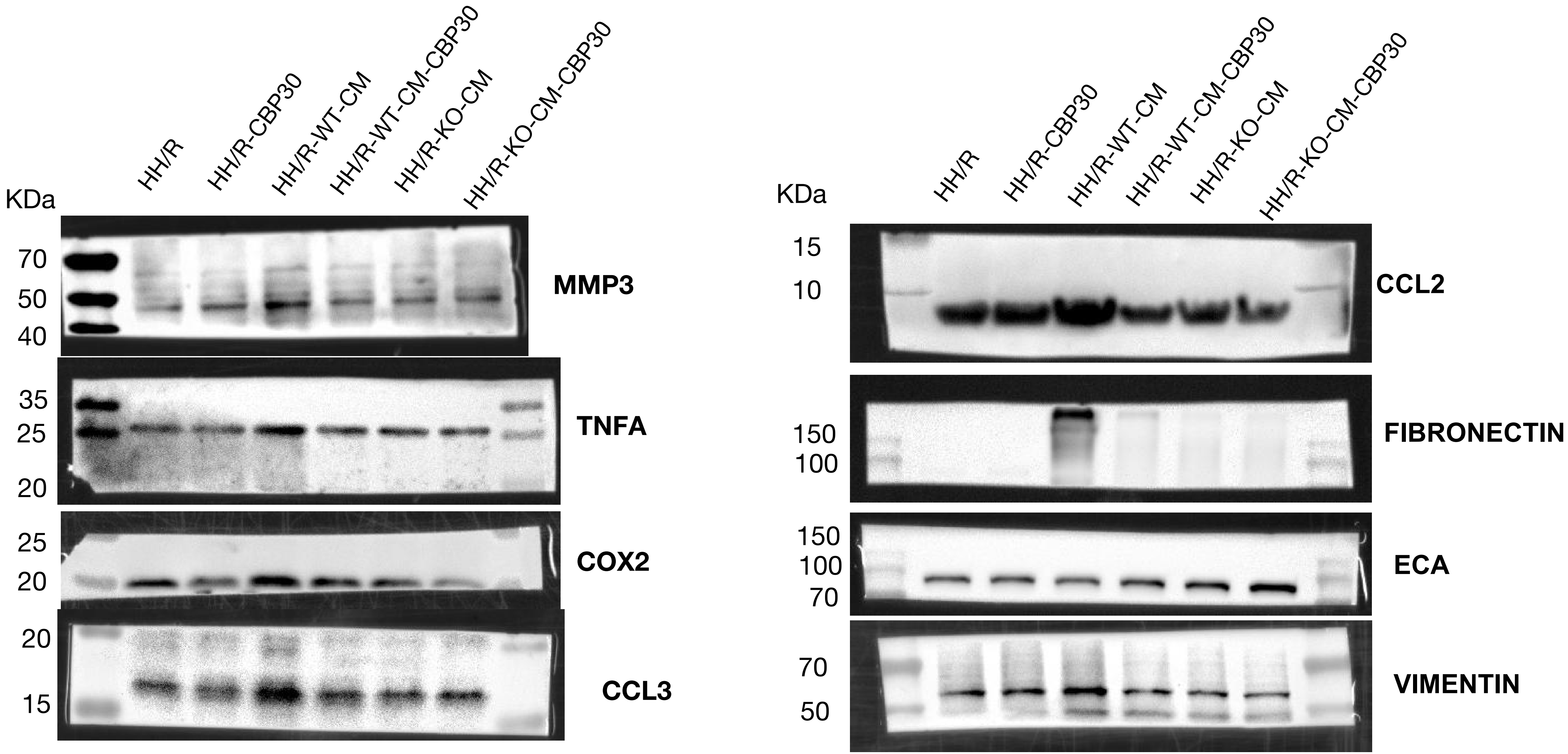

Full unedited gel for Figure 12F

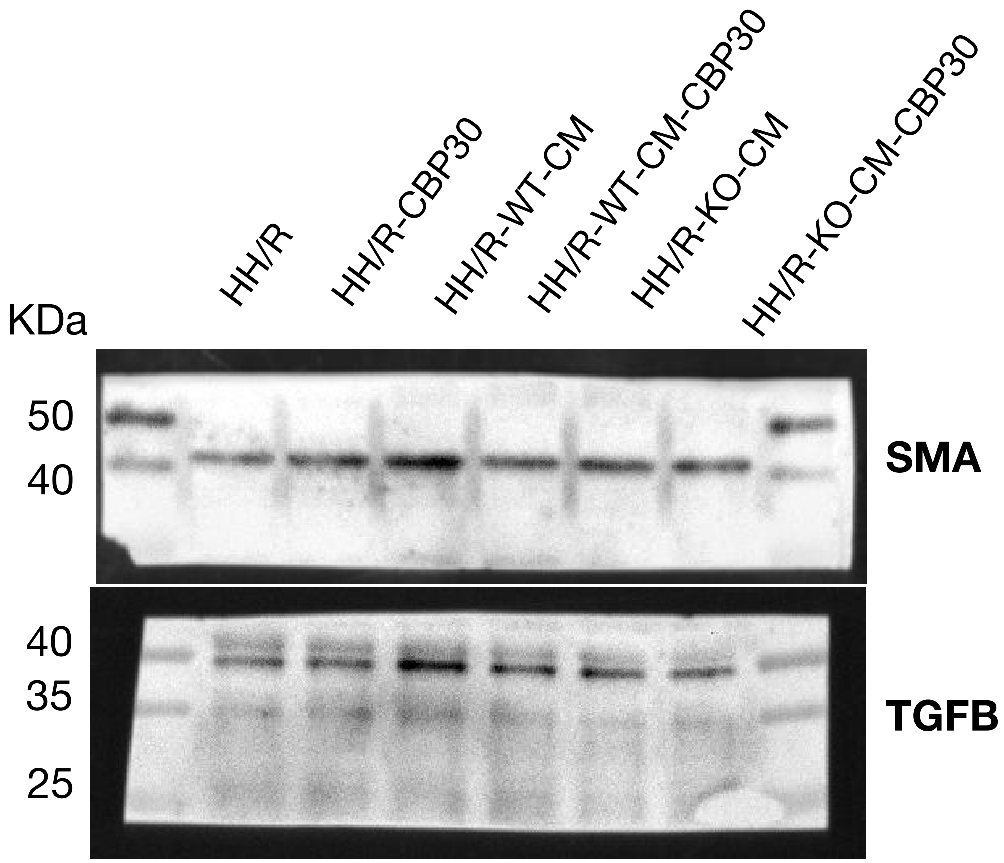

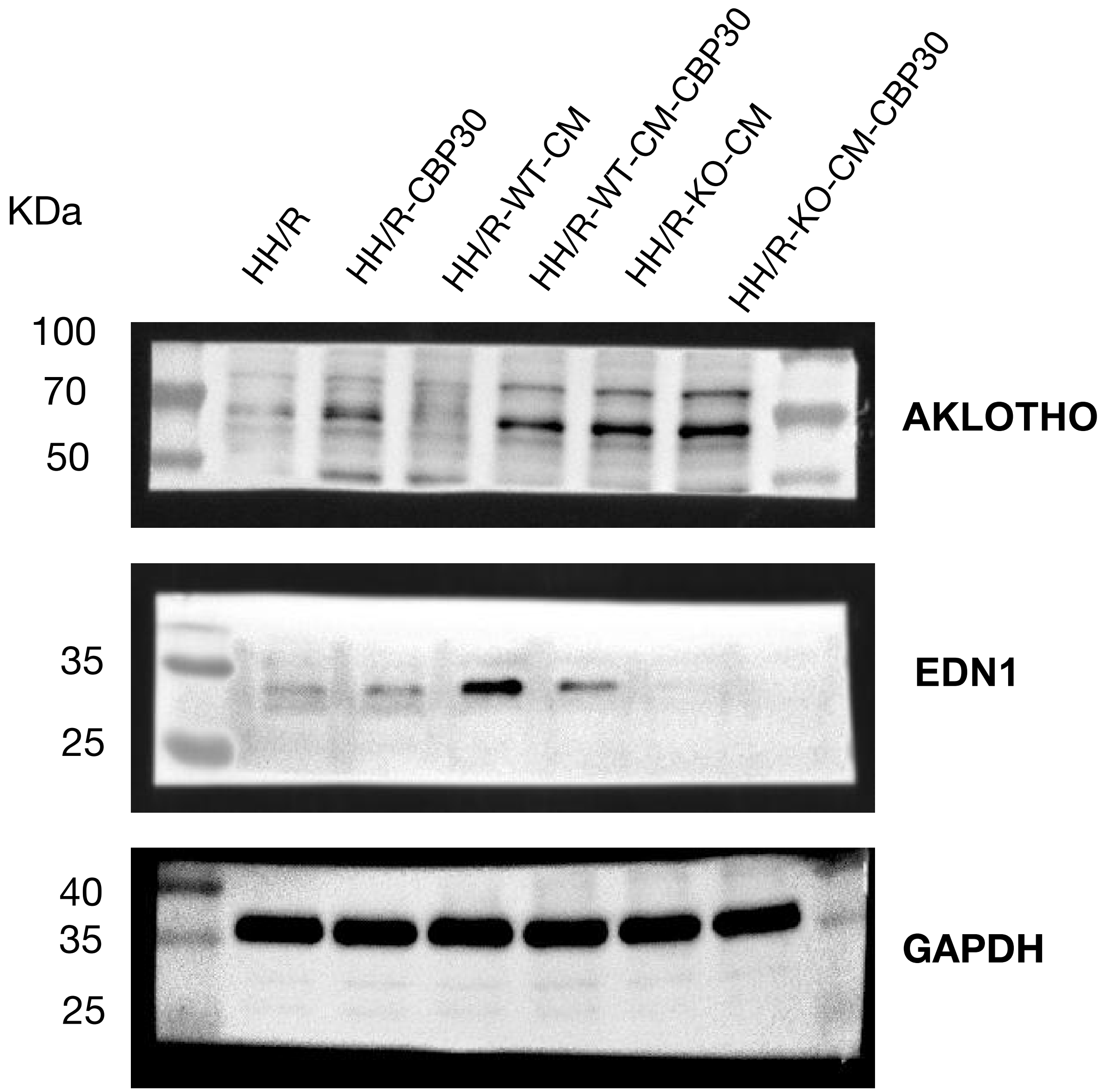

Full unedited gel for Supplemental Figure 4A

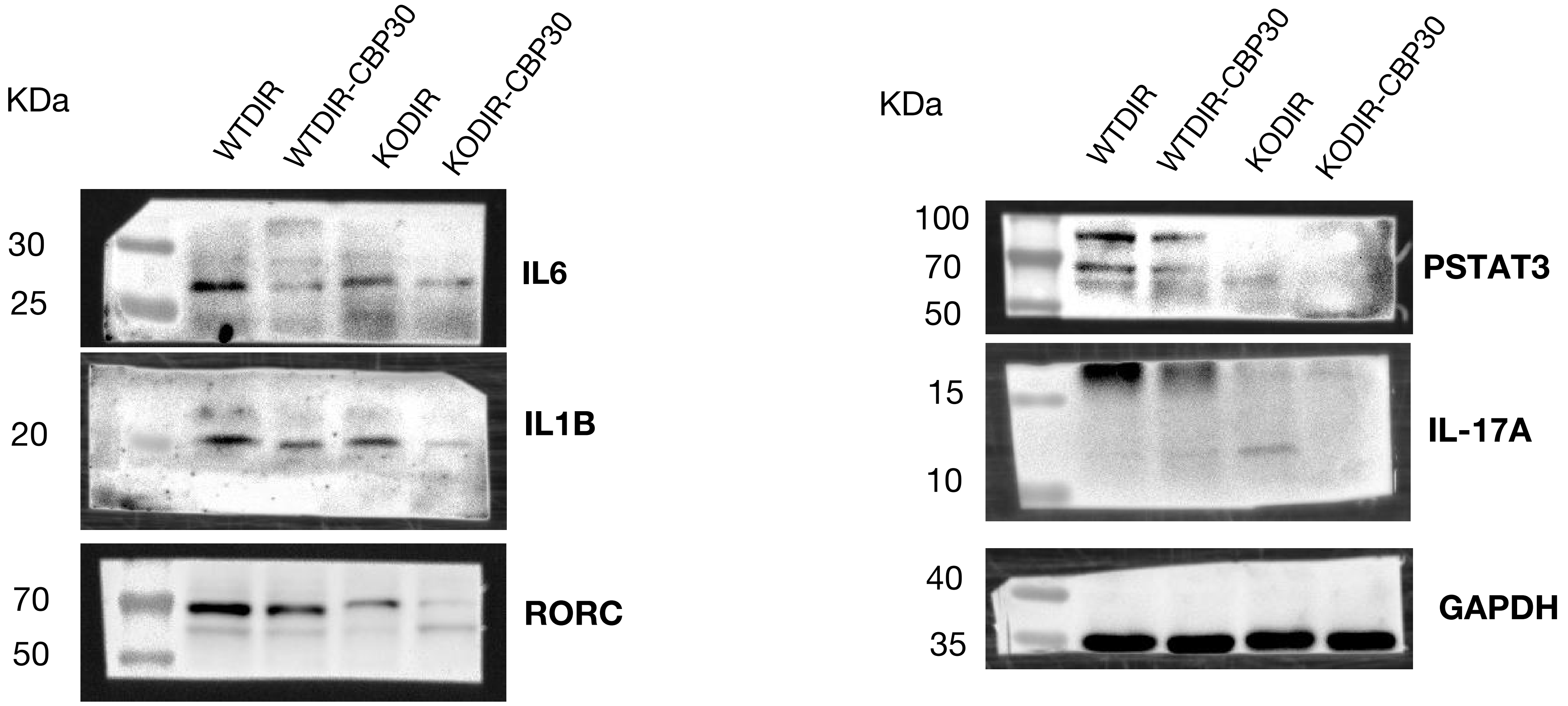

Full unedited gel for Supplemental Figure 4C

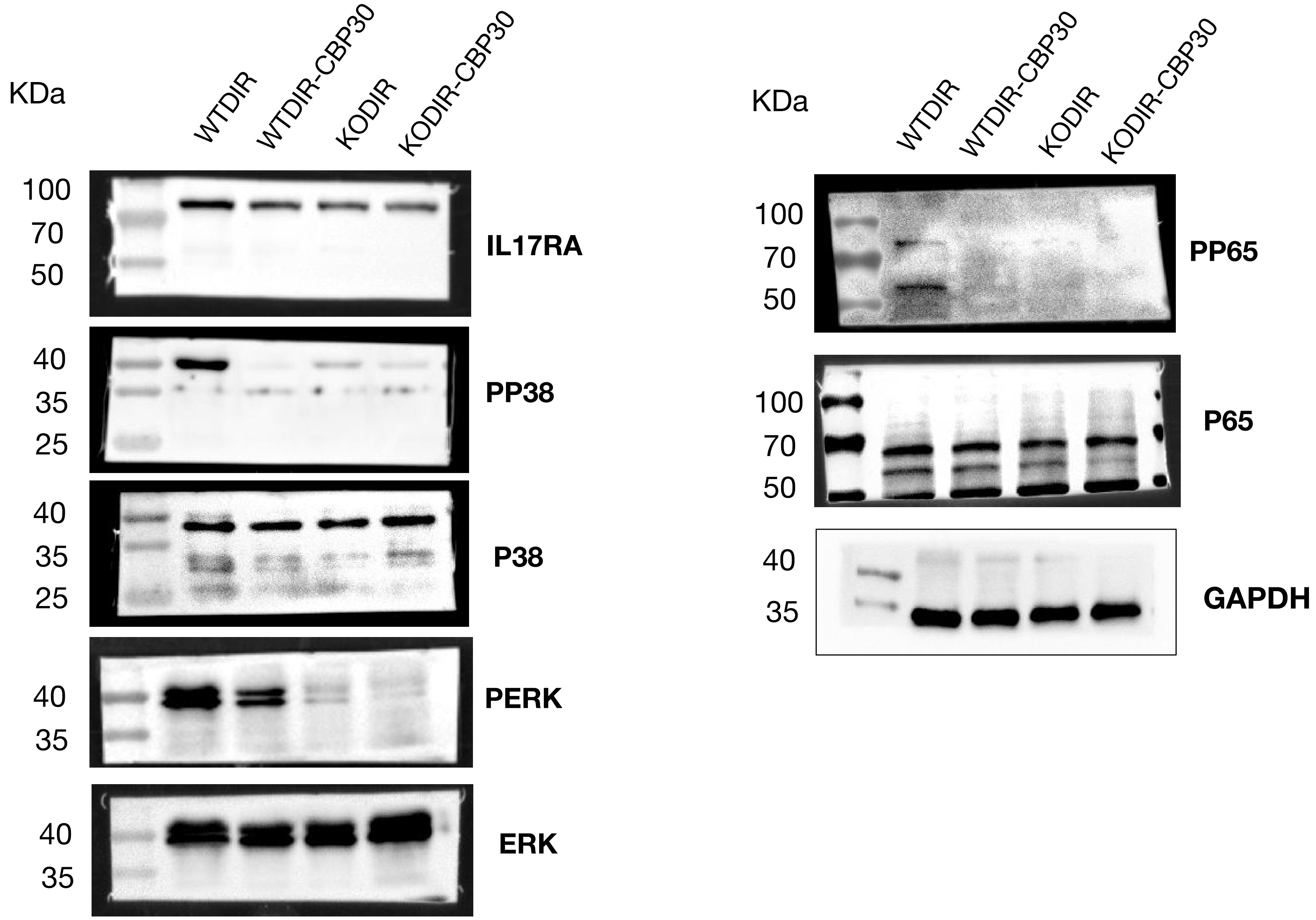

Full unedited gel for Supplemental Figure 4E

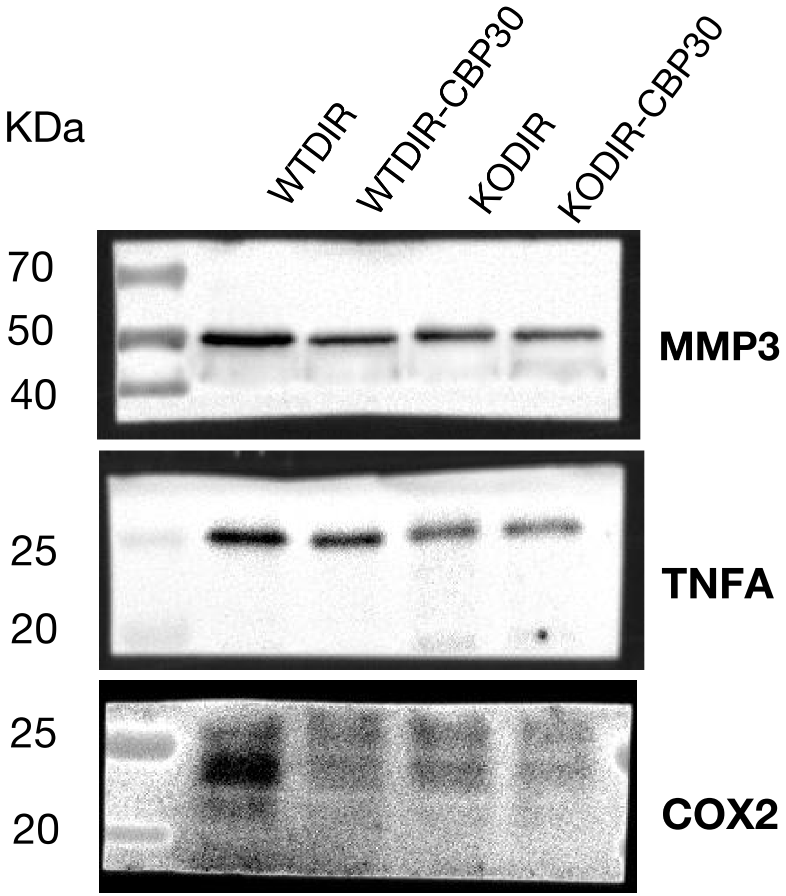

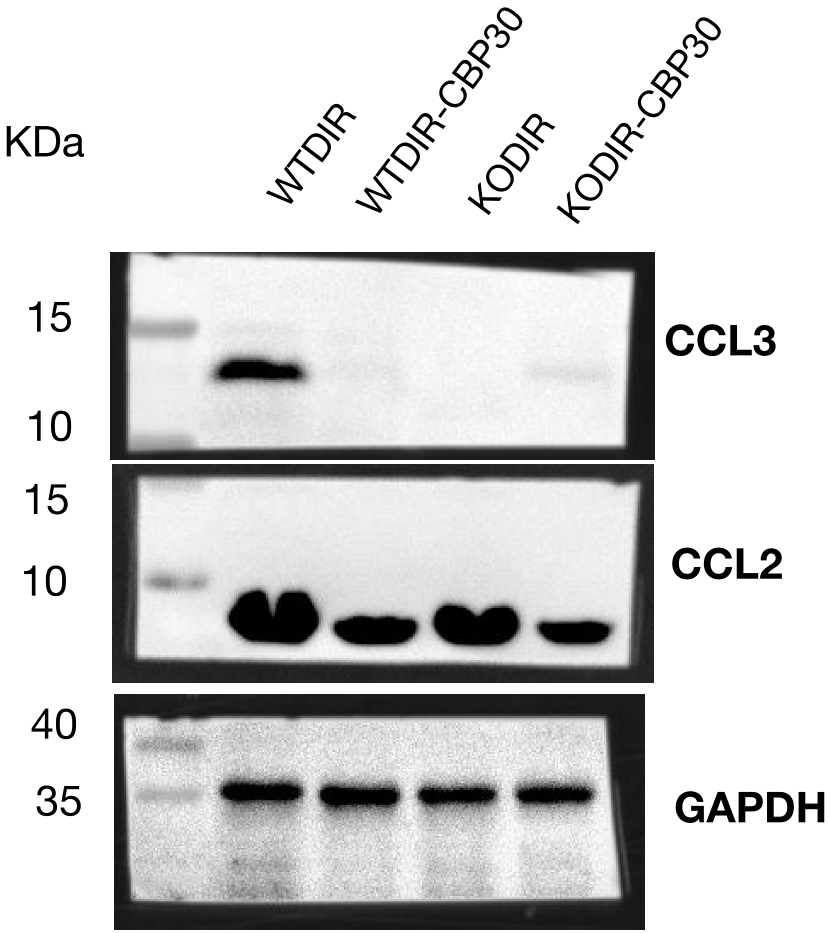

Full unedited gel for Supplemental Figure 4G

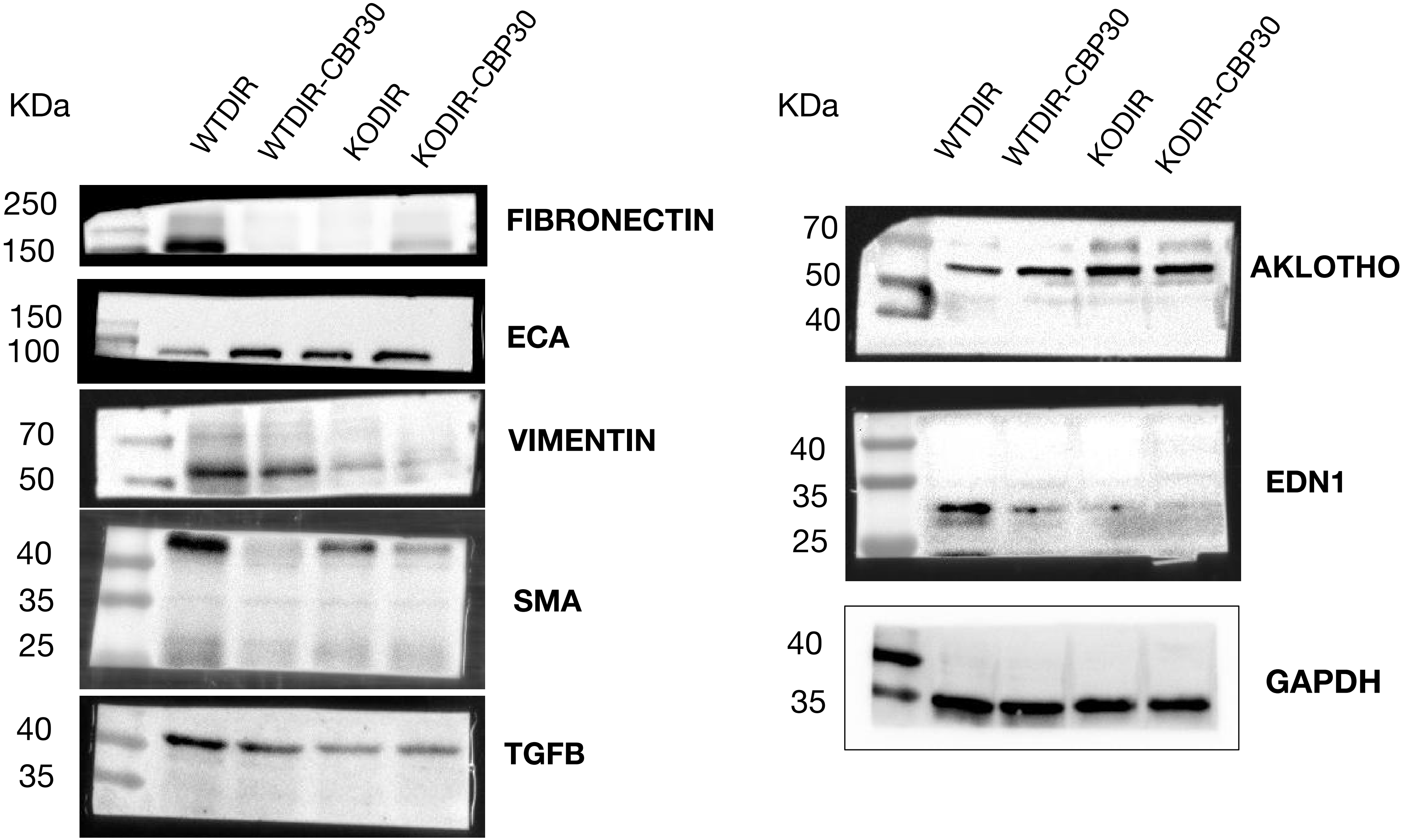

Supplement: Unedited blot and gel images [file jciinsight-10-187653-s135.pdf]
